# Targeting ALG3/FOXD1/BNIP3 Axis Prevents Mitophagy and Gemcitabine Resistance of Nasopharyngeal Carcinoma

**DOI:** 10.7150/ijbs.101585

**Published:** 2025-02-10

**Authors:** Zhanwang Wang, Yi Jin, Dong He, Yuxing Zhu, Mengqing Xiao, Xiaoming Liu, Yaxin Cheng, Ke Cao

**Affiliations:** 1 Department of Oncology, Third Xiangya Hospital of Central South University, Changsha, 410013, China.; 2 Department of Radiation Oncology, Hunan Cancer Hospital, The Affiliated Cancer Hospital of Xiangya School of Medicine, Central South University, Changsha, China.; 3 Central South University, Changsha, 410013, China; 4 Department of Gastroenterology, Third Xiangya Hospital of Central South University, Changsha, 410013, China.

**Keywords:** Nasopharyngeal Carcinoma, Mitophagy, FOXD1, ALG3, Gemcitabine

## Abstract

Understanding the specific role and underlying mechanisms of mitophagy may provide therapeutic benefit to patients with nasopharyngeal carcinoma (NPC). Forkhead box D1 (*FOXD1*), is overexpressed in NPC. However, its roles in NPC progression and therapy resistance remain largely unknown. NPC tissues displayed increased *FOXD1* expression compared to paired non-tumor tissues, which correlated with worse overall survival (OS). Upregulation of *FOXD1* promoted NPC cell proliferation, colony formation, migration, invasion, and impaired sensitivity to GEM by enhancing mitophagy levels. Mechanistically, *FOXD1* promoted mitophagy in NPC cells by transcriptionally initiating BNIP3 expression. This enhanced mitophagy, in turn, promoted proliferation, invasion, and migration and reduced NPC cell sensitivity to gemcitabine (GEM). Most interestingly, Asn176 *N*-glycosylation of the FOXD1 protein increased its stability and nuclear localization, thereby transcriptionally activating *BNIP3* expression to promote mitophagy of NPC cells. ALG3 directly interacted with FOXD1 and induced this *N*-glycosylation. Targeting the ALG3/FOXD1/BNIP3 axis offers a promising therapeutic strategy to inhibit the progression of NPC, which highlighting the potential of therapeutics targeting ALG3 and FOXD1 for regulating mitophagy and overcoming GEM resistance.

## Introduction

Nasopharyngeal carcinoma (NPC) is a malignant tumor originating from nasopharyngeal epithelium, with a particularly high incidence rate in Southern China and Southeast Asia 1. The incidence rate in epidemic areas of southern China is reported to be 25-30 per 100,000 population 2. In recent years, the 5-year survival rate of NPC patients has improved to 80%, primarily due to the widespread implementation of intensity-modulated radiotherapy and gemcitabine with platinum (GP regimen) chemotherapy in advanced stages 3, 4. However, the lack of effective predictive markers to identify patients who would benefit from these treatments remains a challenge. While significant progress has been made, the molecular mechanisms of gemcitabine resistance in NPC are still poorly understood. Therefore, identifying effective biomarkers and targets for NPC diagnosis and treatment is crucial.

Forkhead box D1 (FOXD1) is a pivotal member of the evolutionarily conserved forkhead family of winged-helix transcription factors (TFs) 5. Innumerable studies have reported that *FOXD1* performs critical roles in common physiological functions as well as multiple disease progression, such as recurrent pregnancy loss 6, osteoarthritis 7, kidney development 8, and malignant biological progression of several cancers 9-11. However, the expression and biological effect of *FOXD1* in NPC remain largely unknown.

Mitophagy, a specific form of selective autophagy, aims to eliminate damaged mitochondria, preventing the accumulation of damaging mitochondrial DNA (mtDNA) mutations and maintaining cellular quality 12. Dysregulation of mitophagy is implicated in the pathogenesis of numerous human diseases. The most studied and understood regulatory pathway of mitophagy is the PRKN/parkin-PINK1 axis, which is responsible for initiating the clearance of defective mitochondria 13. Furthermore, mitophagy can also be initiated by multiple mitochondrial outer membrane receptors, such as FUNDC1, BCL2L13, BNIP3, and BNIP3L/NIX, which bind to phagophore membranes and facilitate the recruitment of mitochondria for degradation 14.

Glycosylation, a widespread post-translational modification (PTM), is a finely tuned enzymatic reaction process that primarily occurs in the endoplasmic reticulum (ER) and Golgi apparatus 15. It has been established that enzymes can add sugar chains (*N*-glycosylation and *O*-glycosylation) to proteins and lipids 16. This process can stabilize proteins, ensure their proper positioning within the cell, and facilitate proper protein folding by molecular chaperones 16. Recent evidence suggests that aberrant protein glycosylation plays a crucial role in sustained proliferation signaling, cell death evasion, and chemoresistance in various tumors, including NPC 17, 18. Both mitophagy and glycosylation are essential physiological metabolic processes that could be exploited for developing novel cancer therapies. However, the interplay between these two processes remains largely unexplored. The core structure of N-linked glycans (*N*-glycans) consists of two *N*-acetylglucosamine (GlcNAc) residues, three glucose residues, and nine mannose residues (Glc3Man9GlcNAc2) 19. These GlcNAc residues are linked to asparagine, with additional saccharides attached depending on whether the glycosylation is of the high-mannose, hybrid, or complex type. This process, mediated by various glycosyltransferases known as “asparagine-linked glycosylation” (ALG) enzymes, occurs on dolichol-pyrophosphate carriers 20. Prior to proper folding, proteins carrying the Glc1Man9GlcNAc2 structure are recognized by ER chaperones, calnexin, and calreticulin. Subsequently, aspartate-linked glycosylation 3 (ALG3; α-1,3-mannosyltransferase) catalyzes the first mannosylation step on the lipid-linked Man5GlcNAc2 precursor 21.

Herein, we identified *FOXD1* and *ALG3* to be upregulated in NPC and correlated with poor prognosis. *In vitro* and *in vivo* assays using loss-of-function and gain-of-function approaches confirmed that *FOXD1* could promote NPC growth and metastasis. The oncogenic role of *FOXD1* was attributed to its transcriptional regulation of *BNIP3* expression, which further activated BNIP3-mediated mitophagy in NPC cells. Notably, this study demonstrated that ALG3-mediated *N*-glycosylation of FOXD1 could enhance its nuclear localization, thereby promoting BNIP3-mediated mitophagy and reducing the sensitivity of NPC cells to gemcitabine. Targeting ALG3 in NPC with high *FOXD1* expression prevented mitophagy and enhanced the anti-tumor activity of gemcitabine. Overall, this finding provides a novel strategy to improve the clinical therapeutic efficacy of gemcitabine in NPC patients.

## Materials and Methods

### Clinical specimens

This study was approved by the Institutional Review Board of Third Xiangya Hospital of Central South University (2022-S264). Before the study began, written informed consent for participation in the study was obtained from the patients. All paraffin-embedded biopsy tissues were obtained from NPC patients with detailed clinical characteristics. Long-term follow-up data were collected from January 2014 to December 2018.

### Immunohistochemistry (IHC)

After fixing, embedding, sliding, and deparaffinizing, the tissue sections were blocked with 5% BSA and 3% H_2_O_2_. The sections were then incubated with antibodies against FOXD1 (Biorbyt, orb32533), BNIP3 (Abcam, ab109362), ALG3 (Proteintech, 20290-1-AP), Ki67 (Genetex, GTX16667), and Caspase 3 (Proteintech, 19677-1-AP) at 4 °C overnight.

### Cell culture

Normal nasopharyngeal epithelial cell line NP69 and human NPC cell lines (C666-1, CNE-1, 5-8F, HONE1, and HK-1) were purchased from the Center for Advanced Research of Central South University (Changsha, China). NPC cell lines were cultured continuously in RPMI-1640 (Sigma-Aldrich, USA) supplemented with 10% fetal bovine serum (FBS, Gibco, USA). The NP69 cell line was cultured with keratinocyte serum-free medium (KSFM, Invitrogen, USA) containing bovine pituitary extract (BD Biosciences, USA). All cells were incubated in a humidified atmosphere (37 °C) with 5% CO_2_.

### qRT-PCR and RNA sequencing

Total RNA was extracted using a RNeasy Mini kit (Invitrogen, USA), and complementary DNA (cDNA) was synthesized using a PrimeScript™ RT reagent Kit (Takara, Dalian, China). Quantitative real-time PCR (qPCR) was performed with SYBR Green (Takara, Beijing, China) using primers targeting genes. RNA array sequencing was carried out on an Illumina-HiSeq machine using paired-end sequencing with the support of Lc-Bio Technologies (Hangzhou, China). The sequences of primers are shown in **Supplementary Table S1.**

### Western blotting analysis and immunoprecipitation

Cells were washed with PBS and lysed in Radioimmunoprecipitation assay (RIPA) buffer containing a phosphatase and protease inhibitor cocktail (Roche, Basel, Switzerland). Western blotting (WB) was performed following established protocols 16. The following primary antibodies were used in this study at the indicated dilutions: anti-FOXD1 (1:500, Biorbyt, orb32533), anti-BNIP3 (1:1000, Abcam, ab109362), anti-ALG3 (1:400, 20290-1-AP), anti-LC3B (1:2000, Abclonal, A7198), anti-P62 (1:1000, Abclonal, A7758), anti-PINK1 (1:500, Abclonal, A11435), anti-VDAC1 (1:500, Abclonal, A19707), anti-MFN1 (1:2000, Ptgcn, 66776-1-Ig), anti-Parkin (1:1000, Ptgcn, 14060-1-AP), anti-p-Parkin (Ser65) (1:1000, CST, #36866), anti-COXIV (1:2000, Ptgcn, 66110-1-Ig), and anti-β-actin (1:2000, Ptgcn, 66009-1-Ig). Immunoprecipitation was performed using protein G-agarose (Millipore, CA, USA), followed by the development of blots with ECL WB reagents.

### Plasmid construction, cell interference, and transfection

Lentivirus vectors encoding FOXD1-shRNA, ALG3-shRNA, BNIP3-shRNA, or control were obtained from Tsingke Biotech Company (Beijing, China). Additionally, plasmids (ALG3-ove, BNIP3-ove, FOXD1-ove, Flag-FOXD1-WT, Flag-FOXD1-N176Q, Flag-FOXD1-N211Q, Flag-FOXD1-N457Q) were designed and purchased from Gene-Chem Company (Shanghai, China). The transfection efficiency, knockdown efficacy of shRNAs, and overexpression efficiency of plasmids were all assessed using qPCR and WB assays.

### Chromatin immunoprecipitation

A chromatin immunoprecipitation (ChIP) assay was performed using a Pierce Magnetic ChIP Kit (Thermo Fisher, USA) following the manufacturer's instructions. Briefly, the assay immunoprecipitated protein-DNA complexes using an anti-FOXD1 antibody. Purified DNA was then analyzed using both agarose gel electrophoresis and a qPCR assay.

### Cell apoptosis and Transwell invasion assay

To assess NPC cell apoptosis, we employed fluorescence-activated cell sorting (FACS) with an Annexin V-FITC/PI staining kit (Mbchem). Cell invasion was evaluated using a transwell invasion assay. Briefly, 600 μL of medium containing 20% FBS was added to the lower chambers of the transwell insert. Subsequently, 200 μL of serum-free medium containing 5 × 10^4^ NPC cells was plated in the upper chambers pre-coated with Matrigel (BD Biosciences). Following a 24-hour incubation, cells that penetrated the Matrigel and migrated through the upper chamber were fixed, stained with crystal violet, and visualized using an inverted microscope.

### Cell proliferation and colony formation assays

To assess cell proliferation, 1000 cells were seeded per well in a 96-well plate and incubated according to the manufacturer's instructions. Cell viability was then measured using a CCK-8 assay kit (Biosharp, Hefei, China). Colony formation was evaluated by plating 500 cells per well in a 6-well plate and culturing them for two weeks. Colonies were subsequently fixed with paraformaldehyde, stained with crystal violet, and analyzed.

### Dual-luciferase reporter assay

Cells were transiently co-transfected with plasmids containing the reporter of interest and a firefly luciferase reporter construct. Following 48 hours of transfection, luciferase activity was measured using the Steady-Glo® Luciferase Assay System (Promega) to assess both reporter and firefly luciferase expression. Firefly luciferase activity was then normalized to Renilla luciferase activity (often included in the co-transfection system) to control for variations in transfection efficiency, providing a more accurate reflection of reporter gene expression.

### Immunofluorescence staining

Cells were seeded onto polyethyleneimine-coated coverslips. After washing with PBS, they were fixed in paraformaldehyde for 20 minutes, permeabilized with 0.3% Triton X-100 for 15 minutes, and blocked with 3% BSA solution for 1 hour at room temperature. Subsequently, cells were incubated with primary antibodies overnight at 4°C. Following three washes with PBS, they were incubated individually with primary antibodies in 3% BSA for 1.5 hours at 37 °C. Secondary antibodies were then added and incubated for 1 hour in the dark. Finally, nuclei were counter stained with DAPI in PBS for 10 minutes at room temperature. After three washes with PBS (5 minutes each), images were captured.

### Detection of mitochondrial membrane potential

To assess the mitochondrial membrane potential (MMP) of NPC cells, a JC-1 staining kit (Beyotime, Beijing, China) was employed. Briefly, NPC cells were harvested and washed three times with PBS. The cells were then incubated with JC-1 dye (5 μg/ml) at 37°C for 20 minutes. Following three additional PBS washes, the MMP of the cells was evaluated using a BD Accuri C6 Plus flow cytometer. The resulting data was analyzed using FlowJo software.

### Mitophagy staining assay

The level of mitophagy in cells was assessed using a Mitophagy Detection Kit (Dojindo, Japan) following the manufacturer's instructions. This kit includes two key components: Mtphagy Dye, which specifically labels damaged mitochondria, and lyso dye, which stains lysosomes. By combining these dyes, the kit allows for accurate quantification of mitophagy by identifying mitochondria that have been engulfed and degraded by lysosomes.

### Animal experiments

Five-week-old male BALB/c nude mice were randomly assigned to different groups. Each mouse received a subcutaneous injection of 200 µL (1 × 10^6^ cells) of HONE1 cells into the right axillary fossa to establish xenograft tumors. Tumor volume was monitored every 3 days by measuring the longest diameter (length, L) and shortest diameter (width, W) of the tumors. All mice were sacrificed after 25 days, and tumor volume (V) was calculated using the formula V = (L × W^2^)/2.

### Statistical analyses

Statistical analyses were performed using GraphPad Prism (version 9.0; GraphPad Inc., CA, USA). To ensure data robustness, three independent experiments were conducted. A two-tailed unpaired Student's t-test was employed to compare the statistical significance between the two groups, while a one-way analysis of variance (ANOVA) was used to assess the significance for multiple comparisons. A p-value less than 0.05 was considered statistically significant.

## Results

### *FOXD1* was upregulated and associated with poor prognosis in NPC

**Figure 1:**
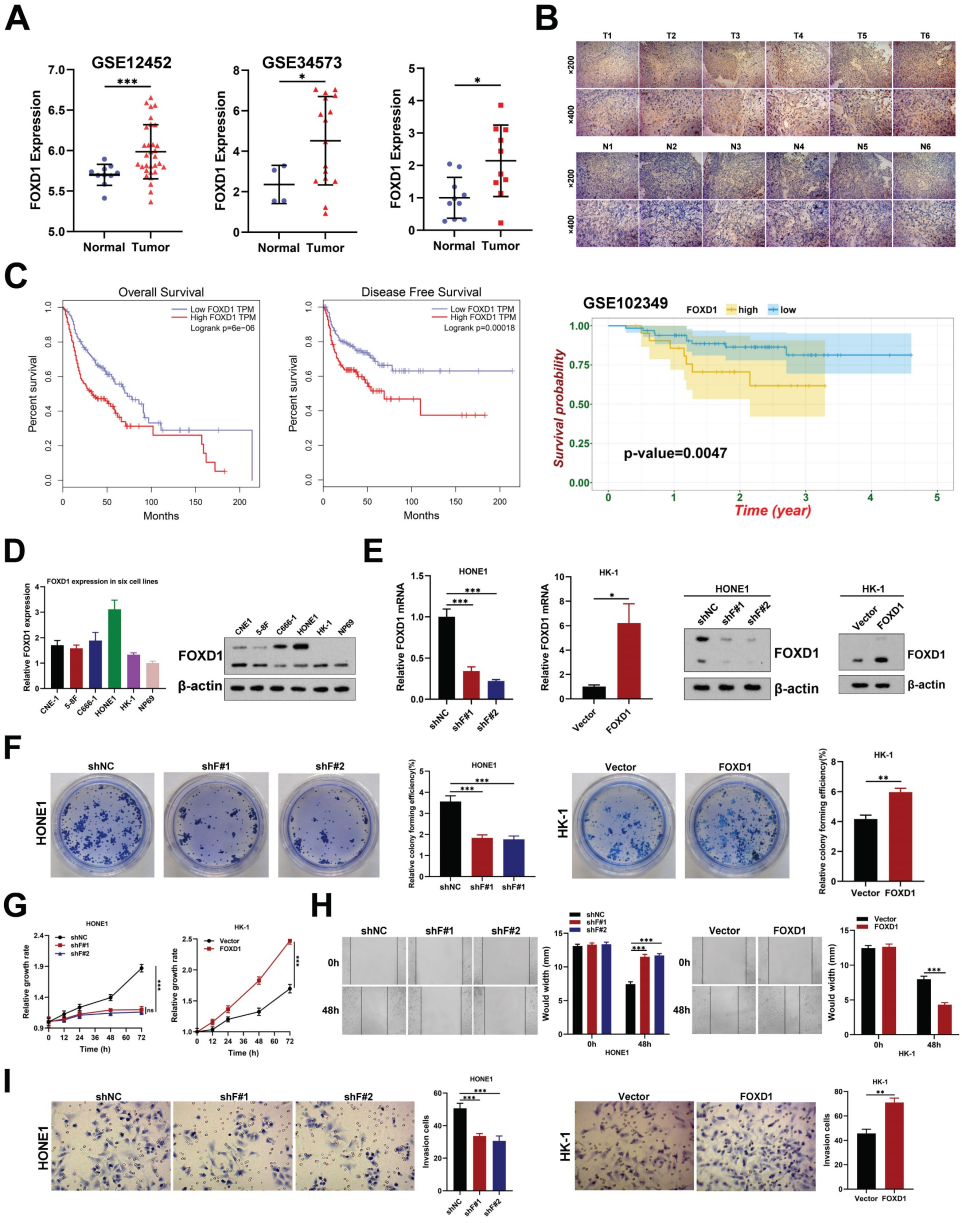
***FOXD1* expression is upregulated in NPC tissues and enhances the proliferation, migration, and invasion of NPC cells. A** Expression level of *FOXD1* mRNA multiple GEO databases (GSE12452 and GSE34573) and that in NPC tumor and normal tissues was detected by qRT-PCR (n=10). **B** Immunohistochemical staining of FOXD1 in NPC tumor and normal tissues. **C** Overall survival and disease-free survival curves of FOXD1 in patients with head and neck tumors by analyzing the GEPIA database, and NPC patients with high *FOXD1* expression have poorer OS in NPC GEO dataset (GSE102349). **D** The mRNA or protein expression levels of FOXD1 in NPC cells and normal nasopharyngeal epithelial cells were detected by qRT-PCR or WB assays. **E** The knockout and overexpression efficiency of FOXD1 was detected by qRT-PCR and WB assays. Colony formation assay, CCK8 assay, wounding healing assay and Transwell assay were used to measure the colony forming ability (**F**), cell growth (**G**), migration (**H**), and invasion ability (**I**) after knockdown and overexpression of FOXD1. The data are presented as the mean ± SEM from three independent experiments. *p < 0.05, **p < 0.01, ***p < 0.001.

Several studies have demonstrated the critical roles of FOX family genes in tumor development and progression across various cancers 22. We initially analyzed gene expression data from the GEO database (GSE12452, GSE34573, and GSE102349). This analysis revealed that three FOX genes (*FOXD1*, *FOXK1*, and *FOXM1*) were associated with overall survival in NPC patients (dataset GSE102349) and showed differential expression between NPC tumors and normal tissues (Supplementary** Fig. 1A**). Notably, *FOXD1* has been largely understudied in the context of NPC. Our analysis of the GEO datasets (GSE12452 and GSE34573) indicated that *FOXD1* expression was significantly higher in NPC tissues compared to non-tumor tissues (**Fig. 1A**), a finding consistent with observations in other tumor types (**Supplementary Fig. 1B**). We further validated these findings using qPCR on NPC specimens, demonstrating that *FOXD1* mRNA levels were indeed elevated in tumor (T) tissues compared with non-tumor (N) tissues (**Fig. 1A**). IHC analysis corroborated these results, revealing a marked increase in FOXD1 protein levels within tumor tissues compared to non-tumor samples (**Fig. 1B**). Finally, survival analysis using GEPIA and the NPC dataset GES102349 demonstrated that higher *FOXD1* expression significantly correlated with poorer overall survival (OS) (**Fig. 1C**). Collectively, these findings suggest a potential role for *FOXD1* in promoting NPC progression.

### *FOXD1* enhances the proliferation, migration, and invasion of NPC cells

To investigate the effects of *FOXD1* on the malignant behavior of NPC cells, we first assessed its expression levels. As expected, *FOXD1* expression was demonstrably higher in NPC cell lines compared to the normal nasopharyngeal epithelial cell line NP69 (**Fig. 1D**). We then manipulated *FOXD1* expression using knockdown and overexpression approaches in HONE1 and HK-1 cells, respectively (**Fig. 1E**). Silencing *FOXD1* in HONE1 cells resulted in a significant decrease in their growth and colony formation ability (Fig. 1F, 1G). Conversely, *FOXD1* overexpression in HK-1 cells substantially promoted these same cellular processes (**Fig. 1F, 1G**). Furthermore, *FOXD1* knockdown led to diminished migration and invasion potential of HONE1 cells, while overexpression in HK-1 cells yielded contrasting effects (**Fig. 1H, 1I**). These findings provide compelling evidence that *FOXD1* plays a crucial role in NPC cell proliferation, migration, and invasion *in vitro*.

### Ectopic expression of *FOXD1* induces mitophagy in NPC cells

**Figure 2:**
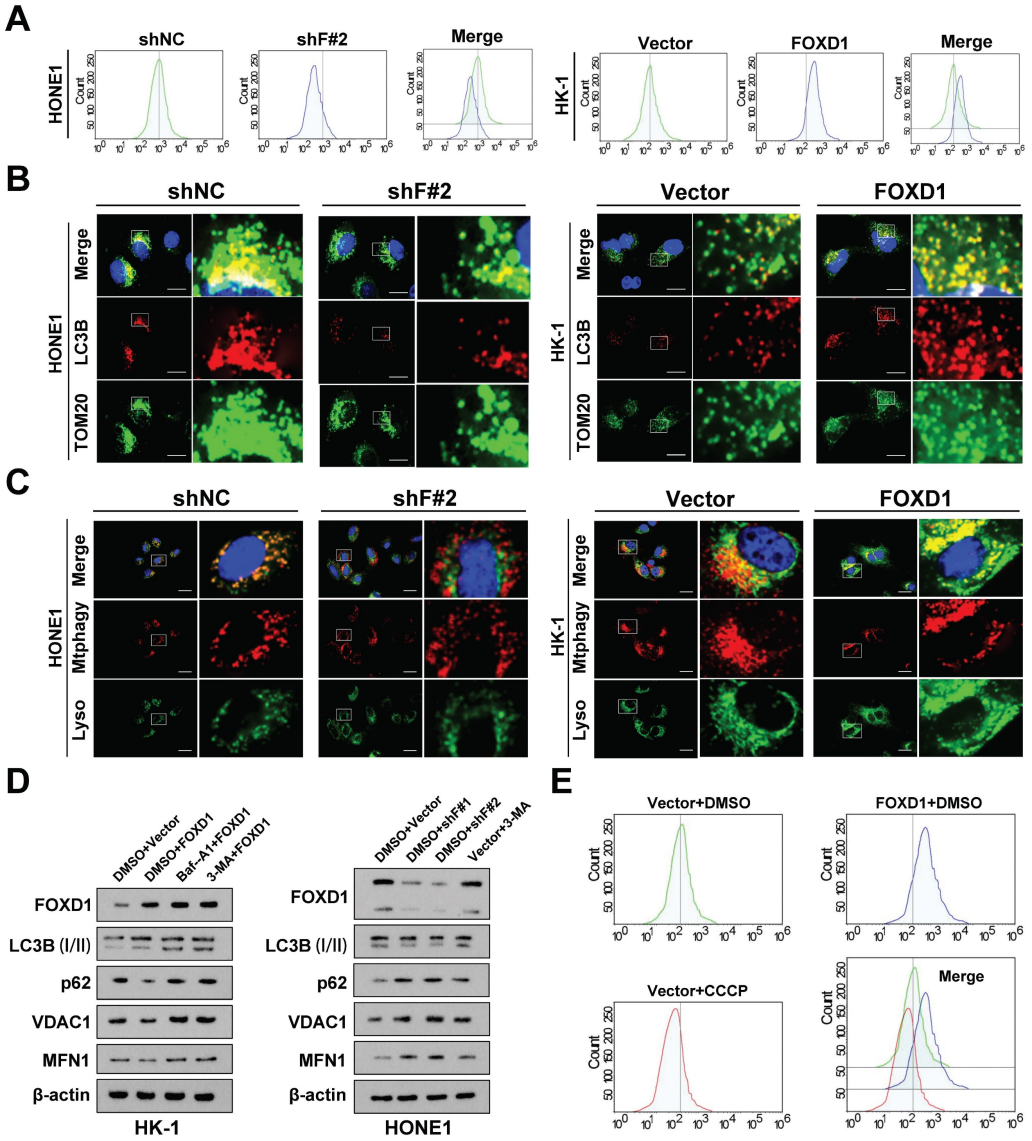
**Ectopic expression of *FOXD1* induces mitophagy in NPC cells. A** The results of JC-1 assay showed that *FOXD1* could increase the membrane potential (MMP) level of HONE1 and HK-1 cells. **B** Fluorescence co-localization experiments showed that TOM20 and LC3B co-localization decreased after *FOXD1* knockdown in HONE1 cells, while TOM20 and LC3B co-localization increased after *FOXD1* overexpression in HK-1 cells. **C** The results of Mtphagy Dye and Lyso Dye showed that mitochondrial and lysosome co-localization decreased after *FOXD1* deletion in HONE1 cells, while mitochondrial and lysosome co-localization increased after *FOXD1* overexpression in HK-1 cells. **D** *FOXD1* increased the expression of autophagy marker LC3B protein and decreased the expression of p62, VDAC1 and MFN1 protein level. **E** The effects of overexpression of *FOXD1* and CCCP on mitochondrial membrane potential of HK-1 cells were detected by JC-1 assay. Scale bars: 10μm.

RNA sequencing was employed to identify downstream signaling pathways and target genes potentially responsible for the anti-tumorigenic effects observed upon *FOXD1* knockdown in NPC cells. The analysis revealed that *FOXD1* knockdown resulted in the downregulation of 509 genes and the upregulation of 609 genes (|Log2FC|>2, p < 0.05). Notably, these differentially expressed genes were significantly enriched in biological processes associated with the regulation of mitophagy and autophagy (**Supplementary Fig. 1A**). To investigate whether *FOXD1* regulates mitophagy in NPC cells, we employed a JC-1 staining assay. This assay indicated that MMP decreased in HONE1 cells following *FOXD1* knockdown, while overexpression of *FOXD1* increased MMP (**Fig. 2A**).

Furthermore, compared to control cells, cells with *FOXD1* overexpression displayed a more diffuse LC3B punctae pattern, suggesting increased autophagosome formation. Additionally, these cells exhibited enhanced co-localization of LC3B with the mitochondrial outer membrane protein TOM20 (**Fig. 2B**), a marker of autophagic sequestration of mitochondria. Finally, co-localization analysis using mitophagy and lysosomal dyes revealed a more pronounced co-localization signal in cells with elevated *FOXD1* expression (**Fig. 2C**). These findings collectively suggest that ectopic *FOXD1* expression enhances mitophagy in NPC cells.

Overexpression of *FOXD1* in HK-1 cells increased the expression of the autophagy marker LC3B (I) and decreased the protein levels of p62, VDAC1, and MFN1. Notably, treatment with the lysosomal inhibitor Bafilomycin A1 (Baf-A1) further increased LC3B expression (**Fig. 2D**). Conversely, inhibiting autophagy using lysosome inhibitors or 3-methyladenine (3-MA) partially reversed this effect. Furthermore, *FOXD1* knockdown (shF#1 and shF#2) in HONE1 cells decreased LC3B expression and increased protein levels of p62, VDAC1, and MFN (**Fig. 2D**). Collectively, these findings suggest that *FOXD1* could promote mitophagy in NPC cells.

### *FOXD1* facilitates the progression and mitophagy of NPC by transcriptionally upregulating *BNIP3 in vitro* and *in vivo*

To further elucidate the mechanism by which *FOXD1* induces mitophagy, we employed a JC-1 staining assay. As expected, treatment with the mitochondrial uncoupler carbonyl cyanide 3-chlorophenylhydrazone (CCCP) significantly decreased the MMP of HONE1 cells, while *FOXD1* overexpression increased the MMP level of HK-1 cells (**Fig. 2E**). Interestingly, co-localization of LC3B and TOM20 was enhanced by both *FOXD1* overexpression and CCCP treatment. However, the co-localization fluorescence intensity was stronger in the CCCP treatment group compared to the *FOXD1* overexpression group (**Supplementary Fig. 3A**). In addition, overexpression of *FOXD1* or CCCP treatment could promote the fusion of mitochondria and lysosomes in HK-1 cells by using Mtphagy Dye and Lyso Dye stain, indicating that *FOXD1* overexpression or CCCP treatment could increase the mitophagy level of HK-1 cells (**supplementary Fig. 3B**). To verify whether *FOXD1* induces mitophagy in NPC cells through the classic PINK1/Parkin pathway or receptor-mediated pathway. Real-time PCR results revealed that *FOXD1* over-expression did not increase the mRNA expression levels of PINK1 and Parkin (**Supplementary Fig. 3C**). Western blot analysis of whole-cell lysate (WCL), cytoplasmic (CYTO), and mitochondrial (MYTO) protein fractions from NPC cells corroborated these findings. Protein expression levels of PINK1, Parkin, and phosphorylated Parkin (p-Parkin) were all upregulated to varying degrees in CCCP-treated cells across all fractions (WCL, CYTO, MYTO). In contrast, *FOXD1* overexpression had no effect on the protein expression levels of these same markers (**Supplementary Fig. 3D**). These results collectively suggest that the canonical PINK1/Parkin pathway is not involved in FOXD1-mediated mitophagy in NPC cells.

**Figure 3:**
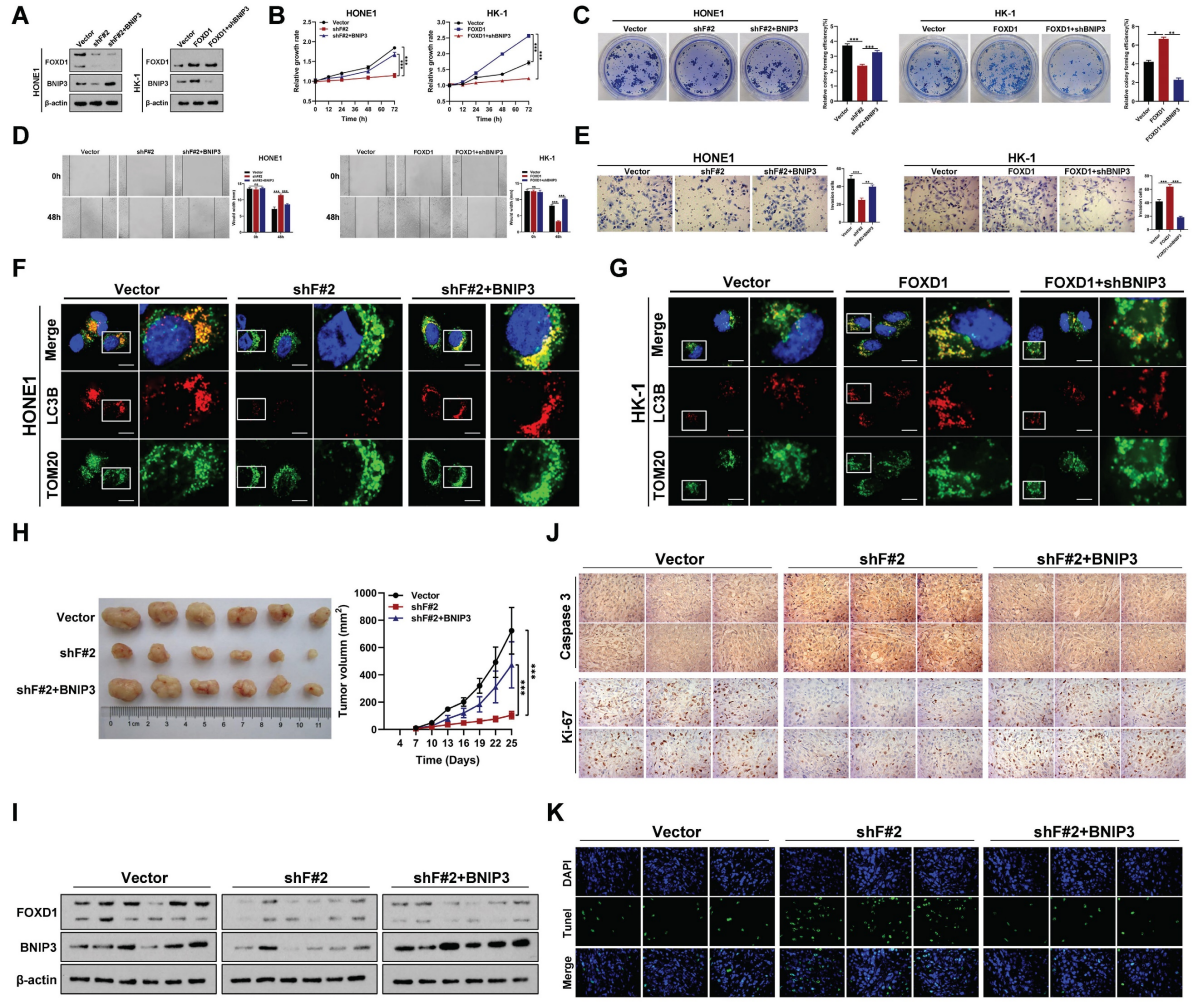
***FOXD1* promotes the progression and mitophagy of NPC through upregulating BNIP3 *in vitro* and *in vivo*. A** The expression levels of FOXD1 and BNIP3 in control group (Vector), FOXD1 knockdown group (shF#2), and FOXD1 knockdown with BNIP3 simultaneous overexpression group (shF#2+BNIP3) were detected by WB. CCK8 assay, colony formation assay, wounding healing assay, and Transwell assay were used to measure the cell growth (**B**), colony forming (**C**), migration (**D**), and invasion (**E**) ability after *FOXD1* knockdown with or without *BNIP3* simultaneous overexpression in HONE1 and HK-1 cells. Localization of LC3B and TOM20 in HONE1 cells after transfection with Vector, shF#2 and shF#2+BNIP3 by fluorescence co-localization assay in HONE1 (**F**) and HK-1 (**G**) cells. **H** Representative tumor images and size of HONE1 tumors formed in the subcutaneous implantation mice was monitored every three days between different groups. **I** WB of FOXD1, BNIP3 and β-actin in the different group tumors. **J** IHC of Caspase-3 and Ki-67 in the different group tumors. **K** Representative TUNEL staining in subcutaneous tumor tissues of mice was shown. *p < 0.05, **p < 0.01, ***p < 0.001. Scale bars: 10μm.

To further elucidate the mechanism by which *FOXD1* increases mitophagy levels in NPC cells, we examined the RNA sequencing results. We observed that *BNIP3*, a mitophagy-related receptor, was significantly downregulated following *FOXD1* knockdown (**Supplementary Fig. 2B**). This finding suggested that *FOXD1* might influence mitophagy levels by regulating BNIP3 expression. We, therefore, selected BNIP3 for further functional assays. Silencing *FOXD1* significantly reduced both the mRNA and protein levels of BNIP3, while *FOXD1* overexpression led to their upregulation (**Fig. 3A**). Furthermore, a ChIP assay demonstrated that FOXD1 could directly bind to the promoter region of *BNIP3* (**Supplementary Fig. 4A-4C**). Analysis of the JASPAR database revealed three potential FOXD1 binding sites within the *BNIP3* promoter region (-2439~-2432bp, -2111~-2104bp, and -653~-646bp). To identify the specific binding sites, we constructed wild-type plasmids (BNIP3-WT), -2439~-2432bp mutant plasmids (BNIP3-MT-2432), -2111~-2104bp mutant plasmids (BNIP3-MT-2104), and -653~-646bp mutant plasmids (BNIP3-MT-646) were constructed. Dual-luciferase reporter assays revealed that compared to the wild-type group, the relative fluorescence intensity was significantly reduced in the BNIP3-MT-2104 and BNIP3-MT-646 groups (**Supplementary Fig. 4D**). These results suggest that the -2111~-2104bp and -653~-646bp regions within the *BNIP3* promoter likely represent binding targets for FOXD1. We further analyzed the GEO datasets (GSE12452 and GSE40290) and found a significant positive correlation between *FOXD1* and *BNIP3* mRNA expression levels (**Supplementary Fig. 5**). Additionally, these datasets revealed high *BNIP3* expression in NPC tissues (GSE12452, GSE40290, and GSE53819) (**Supplementary Fig. 6**). To investigate the functional role of BNIP3 in NPC cells, we constructed *BNIP3* knockdown (shBNIP3) and overexpression (BNIP3) plasmids. qPCR and Western blot assays confirmed the successful manipulation of BNIP3 expression (**Supplementary Fig. 7A, 7B**). Subsequent experiments demonstrated that BNIP3 could promote proliferation, colony formation, migration, and invasion of NPC cells (**Supplementary Fig. 7C-7F**). Moreover, we explored whether *FOXD1* promotes the progression and mitophagy of NPC cells through up-regulating BNIP3. The expression of *BNIP3* significantly increased following *FOXD1* overexpression, while the expression of *BNIP3* was markedly down-regulated with *BNIP3* knockdown (**Fig. 3A**). FOXD1 overexpression significantly increased *BNIP3* expression (**Fig. 3A**), while *BNIP3* knockdown markedly reduced it. *In vitro* experiments demonstrated that *FOXD1* overexpression enhanced growth, cloning, migration, and invasion of NPC cells, and *FOXD1* knockdown suppressed these abilities. Notably, *BNIP3* silencing or overexpression could rescue the effects of *FOXD1* manipulation (**Fig. 3B-3E**). To investigate whether *FOXD1* regulates mitophagy via *BNIP3*, a dual fluorescence localization assay revealed that *FOXD1* knockdown significantly reduced co-localization of TOM20 and LC3B (**Fig. 3F**), whereas *FOXD1* overexpression increased their co-localization (**Fig. 3G**). Importantly, simultaneous *BNIP3* overexpression could offset the decrease in co-localization caused by *FOXD1* knockdown (**Fig. 3F**), and simultaneous *BNIP3* knockdown could offset the increase in co-localization caused by *FOXD1* overexpression (**Fig. 3G**). The co-localization of mitophagy and lysosome markers in HONE1 cells was significantly reduced by *FOXD1* depletion, indicating a decrease in the cells' mitophagy level. Conversely, *BNIP3* overexpression counteracted the downregulation of mitophagy caused by *FOXD1* silencing (**supplementary Fig. S8**). Similarly, *FOXD1* overexpression in HK-1 cells increased the level of mitophagy, but this increase was counteracted by simultaneous knockdown of *BNIP3* (**supplementary Fig. S8**). These results further support the notion that *FOXD1* promotes mitophagy in NPC cells through upregulation of *BNIP3*. To confirm these findings, we examined the efficacy of *FOXD1* knockdown (shF#2) alone or in combination (shF#2 + BNIP3) on NPC growth in immunodeficient mice bearing orthotopic HONE1 xenograft tumors, compared to a blank control. *FOXD1* silencing markedly inhibited the proliferation ability (**Fig. 3H**) and decreased the expression of both *FOXD1* and *BNIP3* (**Fig. 3I**). However, combining *FOXD1* knockdown with *BNIP3* overexpression could rescue these effects (**Fig. 3H, 3I**). Furthermore, *FOXD1* depletion inhibited tumor growth, decreased Ki67 expression, increased Caspase 3 expression (**Fig. 3J**), and promoted cell apoptosis (**Fig. 3K**). Importantly, combining *FOXD1* knockdown with *BNIP3* overexpression reversed these effects (**Fig. 3J, 3K**). Collectively, these results demonstrate that *FOXD1* facilitates NPC progression and mitophagy by transcriptionally upregulating *BNIP3*, both *in vitro* and *in vivo*.

### Inhibition of FOXD1‑BNIP3-dependent mitophagy sensitizes NPC cells to Gemcitabine treatment

**Figure 4:**
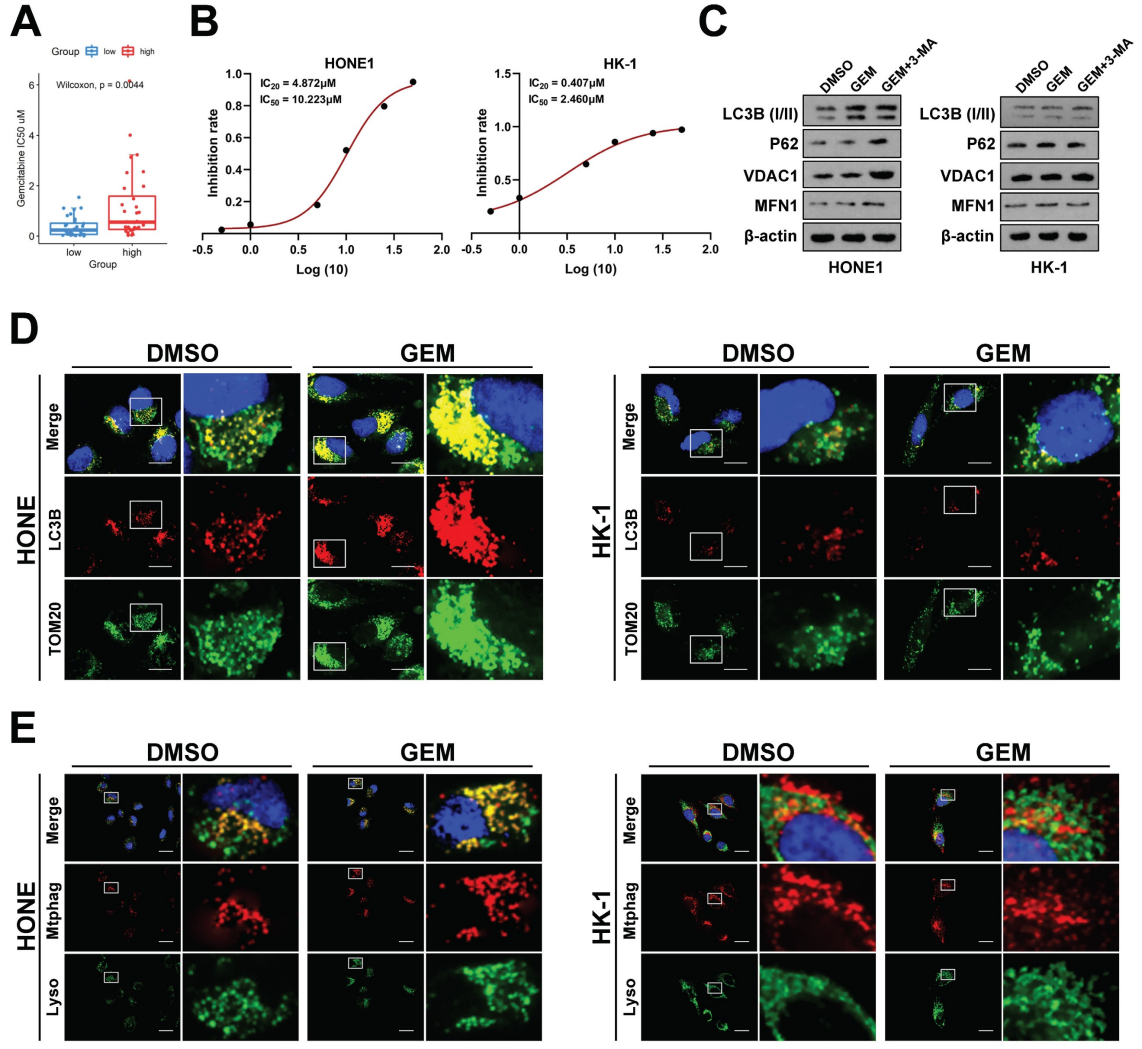
** Gemcitabine could enhance mitophagy level of NPC cells. A** GDSC2 analysis showed that gemcitabine drug sensitivity is negatively correlated with *FOXD1* expression. **B** Inhibition curve of gemcitabine on HONE1 and HK-1 cells.** C** The protein expression levels of mitophagy marker in HONE1 and HK-1 cells treated with DMSO, GEM and GEM+3-MA were detected by WB. The mitophagy level of HONE1 cell treated with DMSO and gemcitabine (GEM) were detected by TOM20 and LC3B dual fluorescence localization assay (**D**) and Mtphagy Dye and Lyso Dye assay (**E**). Scale bars: 10μm.

To further investigate the impact of FOXD1 on NPC drug sensitivity, we employed the GEO dataset GSE102349. Patients were stratified into two groups based on *FOXD1* expression levels: FOXD1-high (37 cases) and FOXD1-low (37 cases). Drug sensitivity prediction analysis was performed using GDSC2 to predict the IC_50_ values of the *FOXD1* high-expression group (high) and *FOXD1* low-expression group (low), respectively, which showed that the drug sensitivity of GEM, the first-line chemotherapy drug for NPC treatment, was most negatively correlated with the expression of *FOXD1* (**Fig. 4A**). Based on this finding, we further explored the role of *FOXD1* in regulating gemcitabine sensitivity and the underlying molecular mechanisms. A Cell Counting Kit-8 (CCK8) assay was performed to assess the IC_20_ and IC_50_ values of gemcitabine in HONE1 and HK-1 cells. The IC_20_ value for HONE1 cells was 4.872 μM, with an IC_50_ value of 10.223 μM. In contrast, HK-1 cells displayed a lower IC_20_ value of 0.407 μM and an IC_50_ value of 2.460 μM (**Fig. 4B**). HONE1 and HK-1 cells were then treated with their respective IC_20_ concentrations of gemcitabine. To assess mitophagy levels, we employed a double fluorescence localization assay for LC3B and TOM20, as well as a Mtphagy and Lyso Dye assay. These assays confirmed that gemcitabine treatment significantly increased the mitophagy level in HONE1 cells (high *FOXD1* expression) but had no effect on the mitophagy level in HK-1 cells (low *FOXD1* expression) (**Fig. 4D, 4E**). To further explore the role of *FOXD1* in gemcitabine-induced mitophagy, HONE1 and HK-1 cells were treated with DMSO (control), GEM, or gemcitabine combined with 3-methyladenine (3-MA), an autophagy inhibitor. Western blot analysis confirmed that gemcitabine treatment in HONE1 cells increased the level of mitophagy, and this effect was inhibited by co-treatment with 3-MA (**Fig. 4C**). However, gemcitabine treatment had no effect on mitophagy in HK-1 cells (**Fig. 4C**). Collectively, these findings suggest that gemcitabine can induce mitophagy in NPC cells with high *FOXD1* expression.

**Figure 5:**
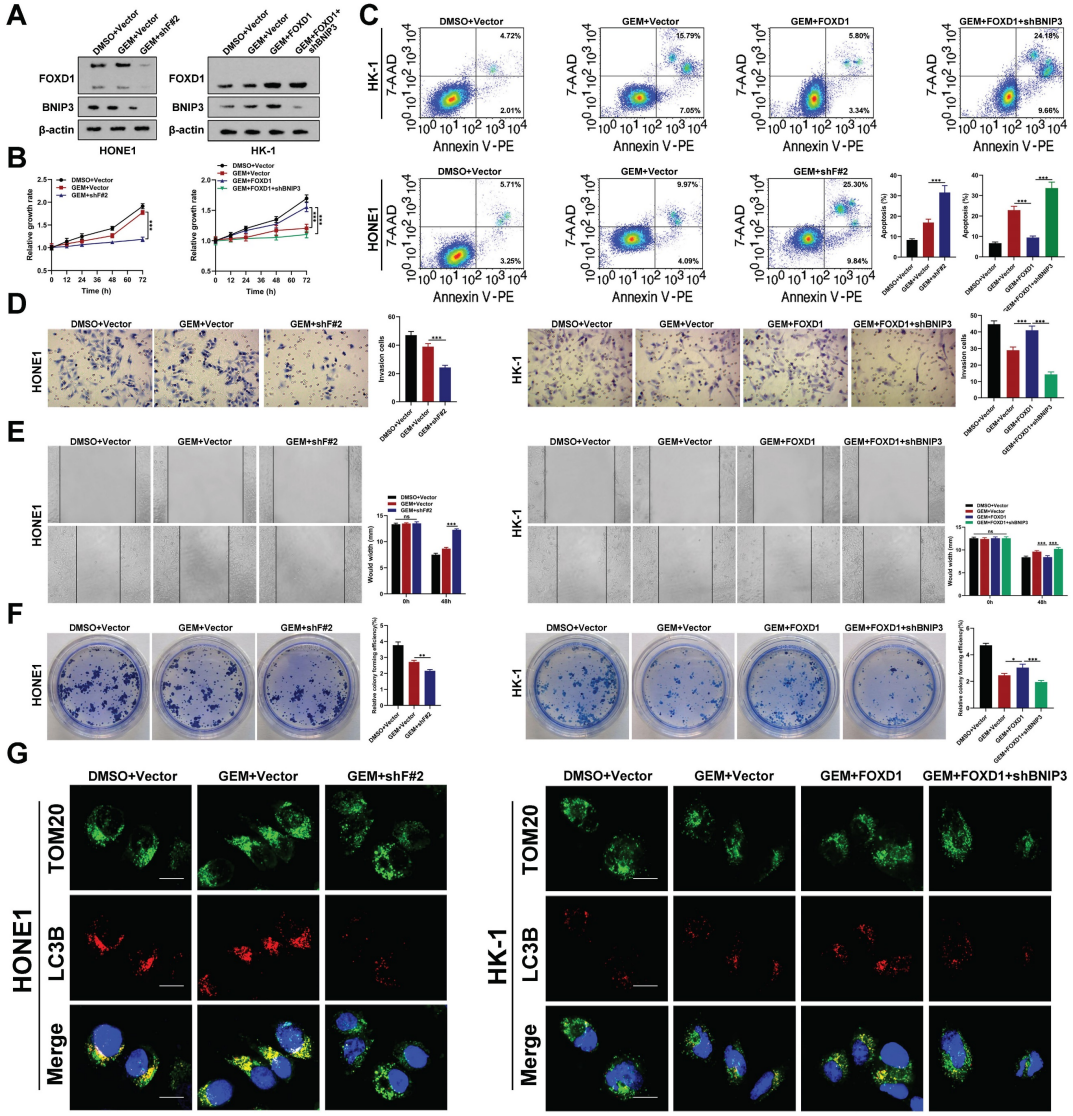
** Inhibition of FOXD1-BNIP3-dependent mitophagy sensitizes NPC cells to Gemcitabine treatment. A** The protein expression levels of FOXD1 and BNIP3 in HONE1 cells after treatment with DMSO+Vector, GEM+Vector and GEM+shF#2 and in HK-1 cells after treatment with DMSO+Vector, GEM+Vector, GEM+FOXD1 and GEM+FOXD1+shBNIP3 by WB assay. CCK8 assay, flow cytometry, Transwell assay, wounding healing assay, and colony formation assay were used to measure the cell growth (**B**), apoptosis (**C**), invasion (**D**), migration (**E**) and colony forming (**F**) ability after treatment with different conditions in HONE1 and HK-1 cells. **G** TOM20 and LC3B co-localization level in HONE1 and HK-1 cells treatment with different conditions were accessed by Fluorescence co-localization experiments. *p < 0.05, **p < 0.01, ***p < 0.001. Scale bars: 10μm.

Western blot analysis revealed that gemcitabine treatment did not significantly alter the protein levels of FOXD1 or BNIP3. However, *FOXD1* overexpression itself increased BNIP3 protein levels (**Fig. 5A**). *In vitro* experiments demonstrated that *FOXD1* knockdown enhanced the inhibitory effect of gemcitabine on HONE1 cell growth (**Fig. 5B**), invasion (**Fig. 5D**), migration (**Fig. 5E**), and colony formation (**Fig. 5F**). Conversely, *FOXD1* overexpression in HK-1 cells mitigated the inhibitory effects of gemcitabine, and this effect could be partially rescued by *BNIP3* silencing. Flow cytometry analysis corroborated these findings, indicating that *FOXD1* reduced the sensitivity of NPC cells to gemcitabine (**Fig. 5C**). To investigate the role of mitophagy in this process, we performed a dual fluorescence localization assay for TOM20 and LC3B. This assay revealed that *FOXD1* knockdown diminished the enhancing effect of gemcitabine on mitophagy in HONE1 cells. In HK-1 cells, gemcitabine treatment alone did not significantly alter the mitophagy level compared to the control group. However, *FOXD1* overexpression could induce mitophagy, and this effect was abrogated by *BNIP3* knockout (**Fig. 5G**). Mtphagy Dye and Lyso Dye staining yielded similar results, demonstrating that *FOXD1* silencing reduced the enhancing effect of gemcitabine on mitophagy levels in HONE1 cells. Likewise, in HK-1 cells, gemcitabine treatment alone had no significant effect on mitophagy, while *FOXD1* overexpression could induce mitophagy, and *BNIP3* depletion could inhibit this effect (**Supplementary Fig. 9**). Taken together, these findings suggest that *FOXD1* promotes mitophagy by upregulating *BNIP3*, thereby reducing the sensitivity of NPC cells to gemcitabine treatment.

### Asn176 N-glycosylation of FOXD1 promotes FOXD1 nuclear translocation

**Figure 6:**
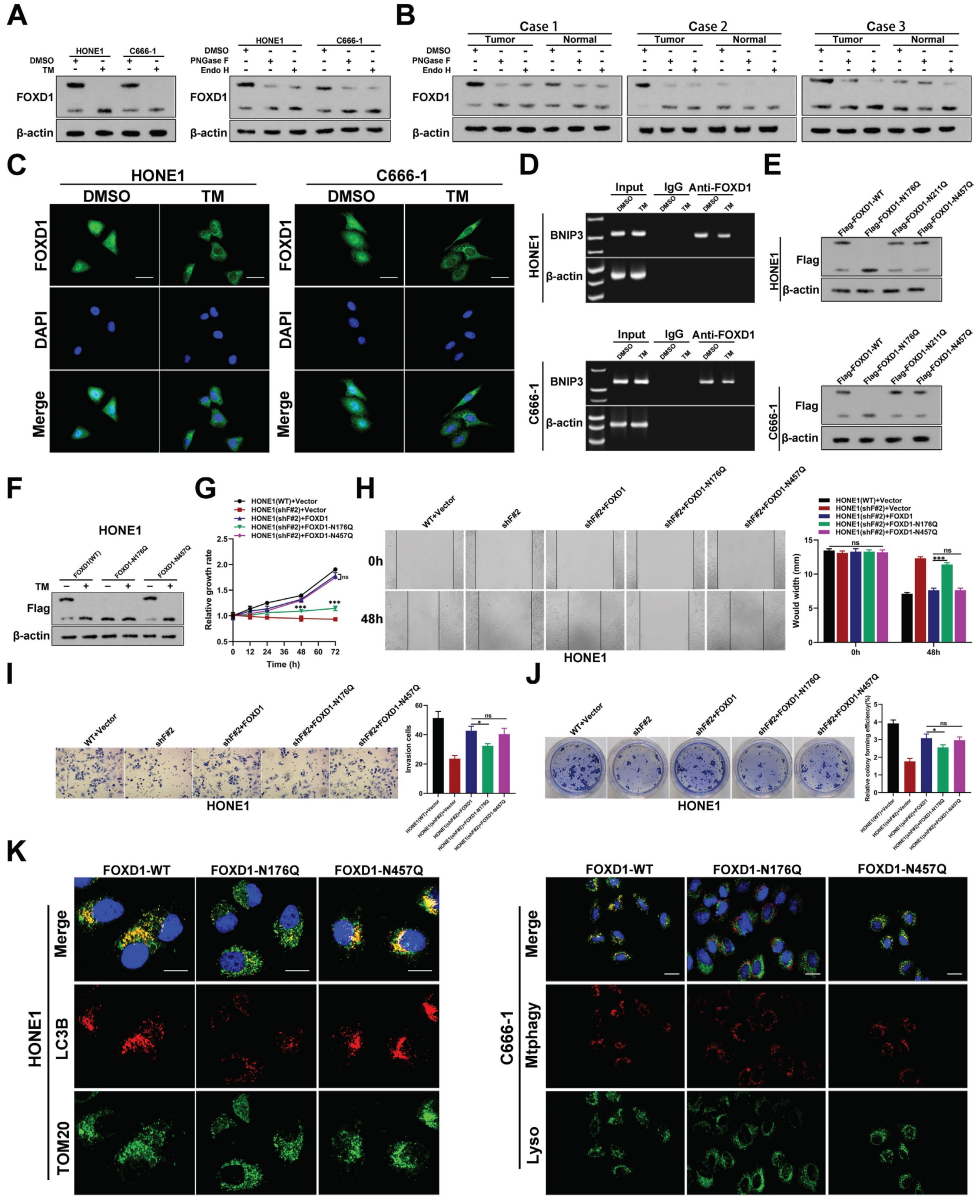
** Asn176 N-glycosylation of FOXD1 promotes its nuclear translocation. A** FOXD1 protein expression was detected by WB with or without TM, Endo H or PNGase F treatment of HONE1 and C666-1 cells. **B** The expression of FOXD1 protein in 3 NPC tissues was detected by WB with or without Endo H or PNGase F treatment of HONE1 and C666-1 cells. **C** Immunofluorescence distribution of FOXD1 in HONE1 and C666-1 cells treated with or without TM. **D** The binding of FOXD1 and BNIP3 promoter region in HONE1 and C666-1 cell treated with or without TM treatment was detected by agarose electrophoresis followed CHIP. **E** WB was used to detect the expression of Flag protein in HONE1 and C666-1 cells transfected with FLAG-FOXD1-WT, FLAG-FOXD1-N176Q, FLAG-FOXD1-N211Q and FLAG-FOXD1-N457Q plasmid. **F** WB detected the bands of Flag protein in HONE1 and C666-1 cells were transfected with different plasmids and treated with or without TM. CCK8 assay, wounding healing assay, Transwell assay and colony formation assay were used to measure the cell growth (**G**), migration (**H**), invasion (**I**), and colony forming (**J**) ability after treatment with different conditions in HONE1 and HK-1 cells. (**K**) Fluorescence of TOM20 and LC3B in HONE1 cell was detected through TOM20 and LC3B fluorescence colocalization experiments after cell was transfected with different plasmids. Scale bars: 10μm.

Western blot analysis detected two distinct FOXD1 protein bands in human NPC cell lines (**Fig. 1D**) and tissues (**Fig. 6B**) with approximate molecular weights of 46 kDa and 60 kDa, respectively. Due to there is a molecular weight double band with such a large difference (46kDa and 60kDa), which made us generally consider glycosylation. Therefore, we hypothesized that the 60 kDa form in NPC cells might represent glycosylated FOXD1, while the 46 kDa form might be non-glycosylated. To further investigate the potential *N*-glycosylation sites of FOXD1 in NPC cells, we consulted online prediction websites such as Glycomine and NetNGlyc. This analysis identified three potential *N*-glycosylation modification sites within the FOXD1 protein sequence (Asn176, Asn211, and Asn457). To verify the presence of *N*-glycosylation on FOXD1 protein, HONE1, and C666-1 cells were treated with tunicamycin (TM), a known *N*-glycosylation inhibitor, at a concentration of 100 ng/mL for 48 hours. Western blot analysis confirmed that TM treatment resulted in a downward shift of approximately 15 kDa in the molecular weight of the FOXD1 protein band (**Fig. 6A**). Furthermore, HONE1 and C666-1 cells were treated with either recombinant peptide-N-glycosidase F (PNGase F) or endonuclease H (Endo H). Compared to the control group, treatment with either PNGase F or Endo H resulted in a near-complete shift of FOXD1 from the 60 kDa band to a band at approximately 46 kDa in both HONE1 and C666-1 cells (**Fig. 6A**). Similarly, Western blot analysis of lysates from clinical NPC tissues revealed a downward shift in the FOXD1 protein bands following treatment with either Endo H or PNGase F (**Fig. 6B**). Taken together, these findings suggest that FOXD1 can undergo *N*-glycosylation. The 60 kDa form detected represents the glycosylated form of FOXD1, while the ~46 kDa form likely represents the non-glycosylated form.

To elucidate the role of *N*-glycosylation in FOXD1 function, immuno-fluorescence (IF) analysis was performed. This assay confirmed that treatment with tunicamycin (TM), an *N*-glycosylation inhibitor, reduced the nuclear distribution of FOXD1 (**Fig. 6C**). Furthermore, ChIP assays combined with agarose gel electrophoresis demonstrated that the transcriptional regulatory effect of FOXD1 on BNIP3 decreased following TM treatment (**Fig. 6D**). These findings suggest that *N*-glycosylation of FOXD1 might promote its nuclear translocation. We consulted online prediction websites such as Glycomine and NetNGlyc to identify potential *N*-glycosylation modification sites within the FOXD1 protein sequence. Based on this analysis, we identified three asparagine (Asn) residues (Asn176, Asn211, and Asn457) as potential *N*-glycosylation sites. To investigate these potential sites, we constructed plasmids harboring mutations at each Asn residue, where the asparagine was replaced with glutamine (Gln). Wild-type plasmid (Flag-FOXD1-WT) and mutant plasmids (Flag-FOXD1-N176Q, Flag-FOXD1-N211Q, and Flag-FOXD1-N457Q) were constructed for subsequent experiments. These plasmids were then transfected into HONE1 and C666-1 cells, respectively. Western blot analysis revealed that compared with wild-type plasmids, only mutations at Asn176 resulted in a decrease in the molecular weight of the FOXD1 protein band. Mutations at Asn211 and Asn457 did not affect the molecular weight (**Fig. 6E**). Further experiments verified that treatment with TM effectively inhibited the *N*-glycosylation modification of FOXD1 and led to a decrease in the molecular weight of the FOXD1 protein band in NPC cells transfected with either the wild-type plasmid (WT) or the mutant plasmid at Asn457. However, TM treatment had no effect on the *N*-glycosylation modification of FOXD1 in NPC cells transfected with the mutant plasmid at Asn176 (**Fig. 6F**). Collectively, these results strongly suggest that Asn176 is the functional N-glycosylation modification site of FOXD1.

To further explore the functional significance of *N*-glycosylation at Asn176, we employed a series of assays. CCK8 and colony formation assays confirmed that FOXD1 knockout significantly reduced the NPC cell growth rate. Overexpression of either the wild-type FOXD1 plasmid (FOXD1-WT) or the FOXD1-N457Q mutant plasmid effectively reversed the inhibitory effect of *FOXD1* silencing on cell growth (**Fig**. **6G, 6J**). In contrast, overexpression of the FOXD1-N176Q mutant plasmid only restored cell growth to a certain extent. Wound healing (**Fig. 6H**) and transwell assays (**Fig. 6I**) revealed that *FOXD1* knockdown markedly reduced the migration and invasion capacities of NPC cells. Overexpression of either FOXD1-WT or FOXD1-N457Q completely and effectively reversed the weakening effect of FOXD1 silencing on these processes. Overexpression of FOXD1-N176Q only restored these abilities to some extent, and the migration and invasion capacities of NPC cells transfected with FOXD1-N176Q were significantly lower than those observed in the FOXD1-WT or FOXD1-N457Q groups (**Fig. 6H, 6I**). In conclusion, *N*-glycosylation at Asn-176 appears to enhance the oncogenic potential of FOXD1. This modification promotes the proliferation, migration, and invasion abilities of NPC cells. Furthermore, while overexpression of FOXD1-WT or FOXD1-N457Q increased the mitophagy level of NPC cells, overexpression of FOXD1-N176Q did not enhance mitophagy.

To investigate whether *N*-glycosylation modification of *FOXD1* affects the sensitivity of NPC cells to GEM, we performed a series of experiments. Western blot analysis revealed a downward shift in the molecular weight of FOXD1 protein bands in the GEM+TM group compared to the GEM group (**Supplementary Fig. 10A**), suggesting that TM treatment inhibited *N*-glycosylation. IF analysis confirmed a significant reduction in FOXD1 nuclear localization and intensity in the GEM+TM group compared to the GEM group (**Supplementary Fig. 10B**). This finding suggests that TM treatment might impair the nuclear translocation of FOXD1. As expected, GEM treatment inhibited the growth rate of NPC cells compared to the control group (DMSO). Notably, the growth rate of cells in the GEM+TM group was significantly slower than that in the GEM group (**Supplementary Fig. 10C**). Flow cytometry results further demonstrated that GEM treatment promoted NPC cell apoptosis compared to the control group. Interestingly, the addition of TM led to an even greater increase in apoptosis (**Supplementary Fig. 10D**). Wound healing, transwell, and colony formation assays all confirmed that GEM inhibited the migration and invasion capacities of NPC cells. Furthermore, the migration and invasion capacities in the GEM+TM group were significantly weaker than those observed in the GEM group (**Supplementary Fig. 10E-10G**). These findings suggest that *N*-glycosylation might contribute to the invasive and migratory abilities of NPC cells. Finally, we investigated the impact of *N*-glycosylation on mitophagy levels. Here, we observed that GEM treatment increased the mitophagy level of NPC cells, while the addition of TM reduced this effect (**Supplementary Fig. 10H, 10I**). In conclusion, *N*-glycosylation plays a critical role in NPC cell sensitivity to gemcitabine treatment. Importantly, *N*-glycosylation can enhance the mitophagy level and reduce the overall sensitivity of NPC cells to gemcitabine.

### ALG3 interacts with FOXD1 and induces its *N-*glycosylation

**Figure 7:**
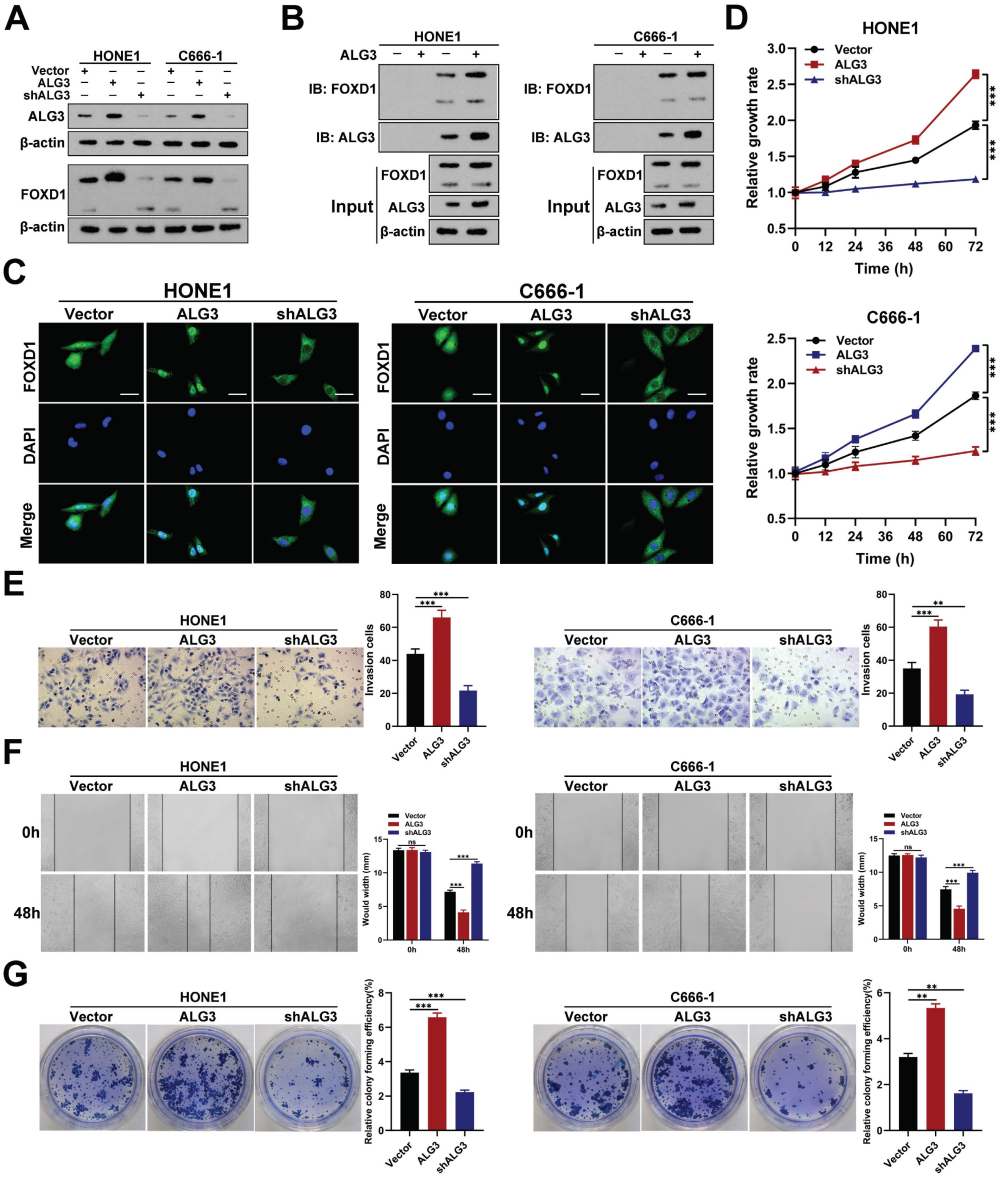
** ALG3 interacts with FOXD1 and induces its *N*-glycosylation. A** Protein expression of ALG3 and FOXD1 in HONE1 and C666-1 cells transfected with different plasmids. **B** co-IP assay verified that FOXD1 could directly bind with ALG3. **C** Immunofluorescence assay indicated that ALG3 could promote the nucleus distribution of FOXD1 in HONE1 and C666-1 cells. CCK8 assay, Transwell assay, wounding healing assay, and colony formation assay were used to measure the cell growth (**D**), invasion (**E**), migration (**F**), and colony forming (**G**) ability after ALG3 silencing or over-expression in HONE1 and HK-1 cells. Scale bars: 10μm.

To identify the glucosyltransferase mediating FOXD1 *N*-glycosylation, we transfected HONE1 cells with a Flag-tagged FOXD1 plasmid. We then performed a co-immunoprecipitation (co-IP) assay using a FLAG-tagged antibody followed by high-performance liquid chromatography-tandem mass spectrometry (HPLC-MS/MS) analysis. This analysis identified alpha-1,3-mannosyltransferase (ALG3) as a potential binding partner for the FOXD1 protein (**Supplementary Fig. 11**). We then investigated the functional role of ALG3 in FOXD1 *N*-glycosylation. Western blot analysis confirmed the knockdown and overexpression efficiency of ALG3. Overexpression of ALG3 resulted in an upward shift in the molecular weight of FOXD1 protein bands, suggesting increased glycosylation. Furthermore, it led to the upregulation of FOXD1 protein expression (**Fig. 7A**). Conversely, ALG3 knockdown resulted in a downward shift in FOXD1 protein bands and a decrease in its expression. Subsequently, ALG3 was knocked down or over-expressed in HONE1 and C666-1 cells following treatment with cycloheximide (CHX), which revealing that ALG3 could strikingly enhance the protein stability of FOXD1 (**Supplementary Fig. 12A**).

Co-IP assays further verified that ALG3 could directly interact with FOXD1 protein in both HONE1 and C666-1 cells (**Fig. 7B**). IF analysis confirmed that ALG3 overexpression increased the nuclear distribution of FOXD1, while knockdown decreased its nuclear localization (**Fig. 7C**). Moreover, WB assay revealed that compared with wild-type plasmids, mutations at Asn176 but not Asn457 in HONE1 and C666-1 cells could affect the the effect of ALG3 on *N*-glycosylation modification of FOXD1 (**Supplementary Fig. 12B**), which indicating that Asn176 is the site that ALG3 mediated the N-glycosylation modification of FOXD1.

These findings collectively suggest that ALG3 mediates *N*-glycosylation modification of FOXD1 and promotes its nuclear translocation. To further explore the role of ALG3 in NPC, we analyzed gene expression data sets from GEO. We found that ALG3 mRNA expression levels were significantly elevated in NPC tissues compared to controls in both GSE12452 and GSE53819 datasets (**Supplementary Fig. 13**). Functional assays demonstrated that ALG3 could significantly promote the proliferation of NPC cells, as evidenced by CCK8 and colony formation assays (**Fig. 7D, 7G**). Furthermore, wound healing and transwell assays revealed that ALG3 enhanced the migration and invasion capacities of NPC cells (**Fig. 7E, 7F**).

### Inhibition of FOXD1‑dependent mitophagy sensitizes NPC cells to GEM treatment

**Figure 8:**
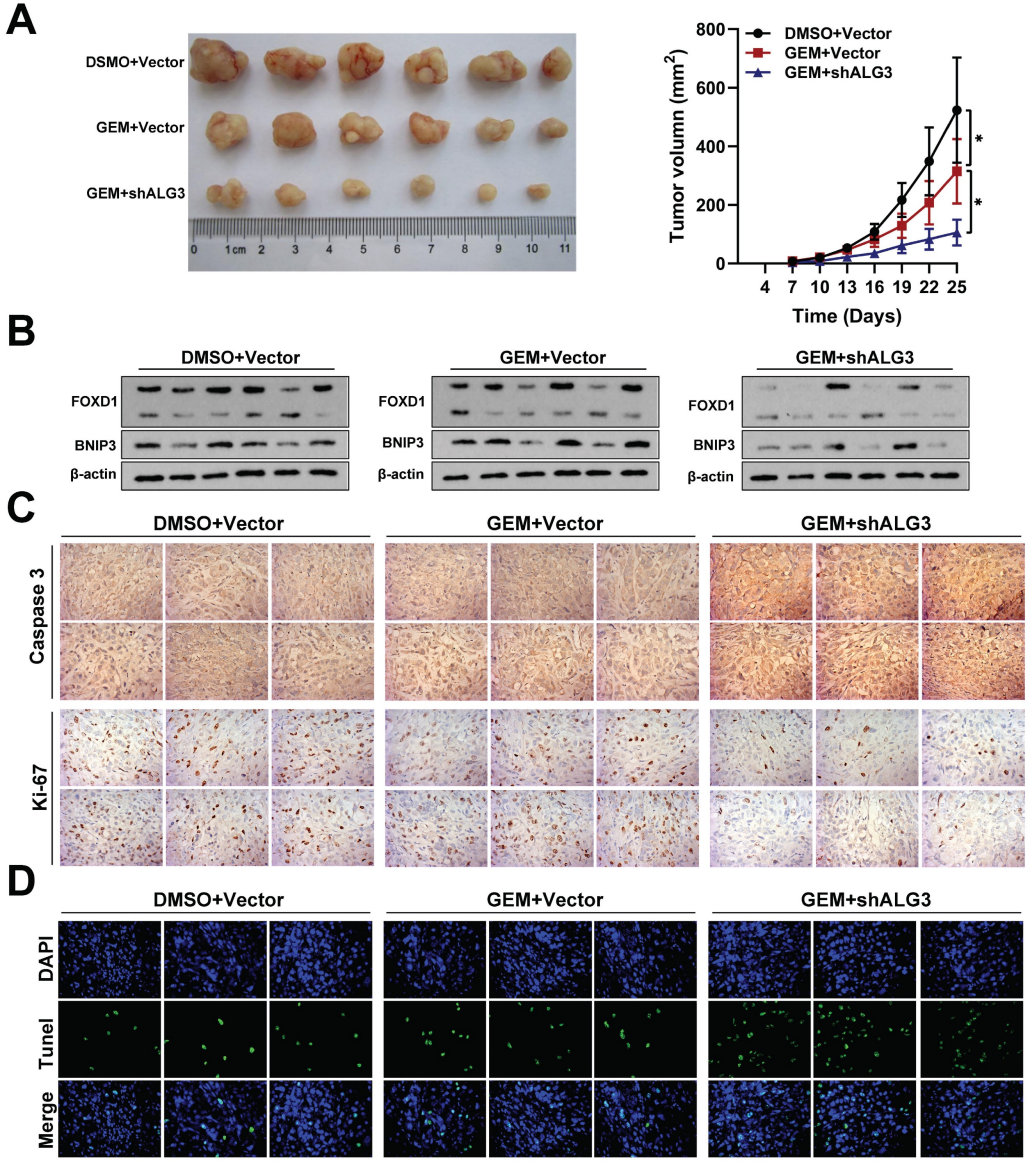
** Inhibition of FOXD1-dependent mitophagy sensitizes NPC cells to GEM treatment. A** Representative tumor images and size of HONE1 tumors formed in the subcutaneous implantation mice was monitored every three days between different groups. **B** WB of FOXD1, BNIP3 and β-actin in the different group tumors. **C** IHC of Caspase-3 and Ki-67 in the different group tumors. **D** Representative TUNEL staining in subcutaneous tumor tissues of mice was shown. *p < 0.05.

To validate our *in vitro* findings, we investigated the efficacy of gemcitabine (5 mg/kg) alone or in combination with shALG3 (gemcitabine 5 mg/kg + shALG3) on NPC growth in immunodeficient mice bearing orthotopic xenograft tumors. A normal saline control group was also included. Gemcitabine treatment alone had minimal effect on the expression levels of FOXD1 and BNIP3, but it did inhibit cell proliferation (**Fig. 8A**). However, the combination treatment with shALG3 depletion led to a significant decrease in the expression of both FOXD1 and BNIP3 (**Fig. 8B**). *In vivo* analysis revealed that both gemcitabine alone and the combination treatment significantly inhibited tumor growth, decreased Ki67 expression and increased Caspase 3 expression (**Fig. 8C**). TUNEL staining further confirmed that both gemcitabine alone and the combination treatment promoted cell apoptosis within the tumors (**Fig. 8D**). These findings support the hypothesis that ALG3 promotes BNIP3-mediated mitophagy in NPC cells by inducing *N*-glycosylation modification and enhancing FOXD1 protein stability.

**Figure 9:**
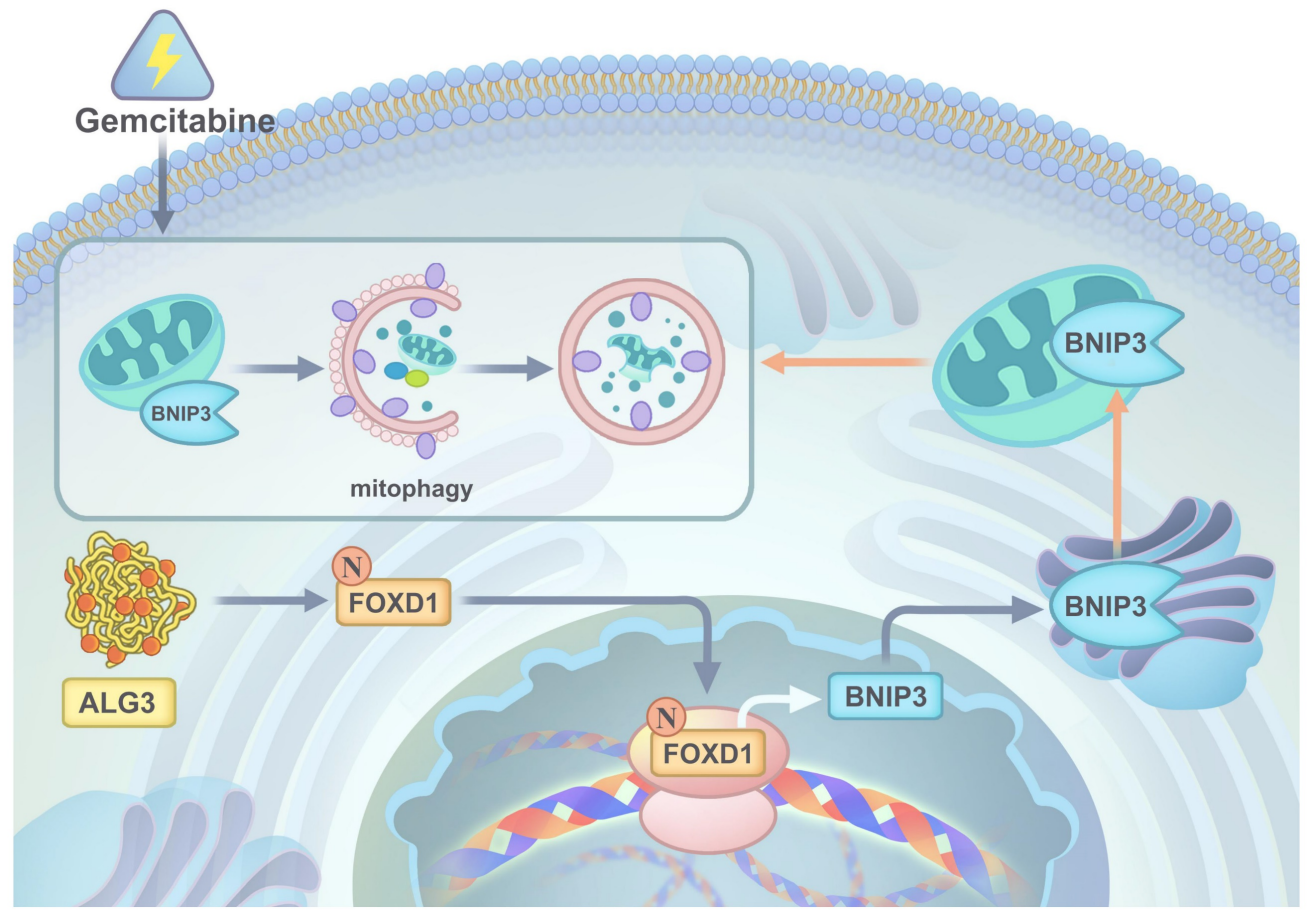
A graphic illustration of ALG3 promoting tumor progression and gemcitabine resistance f NPC by modulating FOXD1 *N*-glycosylation to facilitate its nuclear translocation to enhance BNIP3-dependent mitophagy level.

In conclusion, our comprehensive in *vitro* and *in vivo* studies employing gain-of-function, loss-of-function, and combination therapy approaches have demonstrated the critical roles of the ALG3-FOXD1 axis in mitophagy, a process that contributes to NPC growth and gemcitabine resistance. We found that combining gemcitabine treatment with ALG3 knockdown effectively inhibited NPC cell growth. A schematic representation summarizing the key molecular mechanisms underlying these observations is presented in **Figure 9.**

## Discussion

Mitochondrial flexibility is crucial for the survival of NPC cells under stress conditions such as starvation, hypoxia, and chemo/radiotherapy 23. Mitophagy, the selective degradation of damaged mitochondria, plays a vital role in maintaining cellular health 24. However, growing evidence suggests that mitophagy can also contribute to cancer progression and metastasis 25. Deciphering the factors regulating mitophagy in NPC development remains an area of active research. Transcription factor *FOXD1* has been implicated in the development of various cancers 26-30. Our analysis of GEO datasets revealed that *FOXD1* is among the most significantly upregulated genes in NPC tissues. Herein, we demonstrated that NPC with high *FOXD1* expression exhibits greater malignant potential and a poorer prognosis. Mechanistically, it is highly conceivable that *FOXD1* triggers cytoprotective mitophagy in NPC cells by transcriptionally regulating *BNIP3* expression under cellular stress. Furthermore, we found that *FOXD1* expression negatively correlates with the clinical efficacy of gemcitabine therapy. Interestingly, combining *FOXD1* knockdown or *BNIP3* silencing with gemcitabine treatment, which effectively blocks mitophagy, significantly improved therapeutic outcomes. Collectively, our findings identify *FOXD1* as a novel and crucial factor promoting NPC growth, metastasis, and resistance to gemcitabine therapy through its role in regulating mitophagy. This study highlights *FOXD1* as a potential therapeutic target for NPC treatment.

Mitophagy, the selective degradation of damaged mitochondria, is a cellular response that allows for adaptation to environmental stress 31. Disruptions in mitophagy are increasingly recognized as key contributors to tumor progression 31. Herein, we demonstrated that *FOXD1* functions as an independent prognostic factor and plays a critical role in promoting NPC cell growth, migration, and invasion. Emerging evidence suggests that *FOXD1* expression is associated with aggressive tumor behavior in various cancers. However, the precise role of *FOXD1* in cancer progression remains largely unexplored.

A key question emerging from this work is how *FOXD1* promotes NPC cell growth under stress conditions. RNA sequencing analysis revealed a potential link between *FOXD1* and the autophagy pathway, specifically in BNIP3-mediated mitophagy, which is thought to contribute to NPC growth and metastasis. We hypothesized that FOXD1 might transcriptionally activate* BNIP3* expression, thereby promoting mitophagy and malignant progression in NPC cells, potentially leading to insensitivity to chemotherapeutic drugs. Our findings demonstrated that *FOXD1* overexpression indeed transcriptionally upregulates *BNIP3* expression, suggesting a novel role for *FOXD1* as a regulator of BNIP3-mediated mitophagy. This observation aligns with growing evidence that mitophagy can protect cancer cells from the cytotoxic effects of chemotherapy drugs 32. *BNIP3*, a receptor playing a critical role in mitophagy regulation within cancer cells, is a particularly well-studied example 32. While some studies suggest that BNIP3-mediated mitophagy can induce cell death in specific contexts (e.g., glioma cells treated with ceramides), others have shown a protective role for BNIP3-mediated mitophagy (e.g., SH-SY5Y neuroblastoma cells treated with TNF-α) 32. These findings highlight the context-dependent nature of BNIP3 function, where it can regulate both pro-death and pro-survival mitophagy pathways. Mechanistically, BNIP3 can promote autophagy by activating the autophagy pathway and initiating mitophagy through several mechanisms. First, BNIP3 can inhibit the phosphorylation of mTOR, a key negative regulator of autophagy, either by binding to Rheb or by releasing Beclin-1 through interaction with Bcl-2 33. Second, *BNIP3* can directly initiate the engulfment of damaged mitochondria by autophagosomes. This process relies on the C-terminal transmembrane domain of BNIP3, which allows for its insertion into the outer mitochondrial membrane, as well as its LC3 interaction regions (LIRs) that facilitate binding to LC3B-II, a key autophagic protein 34. In the present study, we observed a significant increase in *BNIP3* expression upon *FOXD1* overexpression. This correlated with enhanced malignant potential of NPC cells, including increased cell growth, migration, and invasion. These findings were further supported by *in vivo* studies. Furthermore, treatment with an autophagy inhibitor or BNIP3 knockdown significantly abrogated the observed enhancement in the malignant potential of NPC cells. Collectively, these findings suggest a novel mechanism by which *FOXD1* promotes NPC progression and chemoresistance. Importantly, FOXD1 can transcriptionally activate *BNIP3* expression, leading to BNIP3-mediated mitophagy and ultimately promoting NPC cell growth, migration, invasion, and insensitivity to chemotherapeutic drugs. Our findings highlight *FOXD1* and *BNIP3* as potential therapeutic targets for NPC treatment.

Gemcitabine remains a cornerstone of first-line therapy for advanced NPC, demonstrably increasing patient survival rates 35. However, real-world clinical data reveals a limited objective response rate, often below 30%, highlighting the emergence of gemcitabine resistance in a significant subset of NPC patients 36. This phenomenon warrants further investigation. Our findings demonstrate that FOXD1 can bind to the *BNIP3* promoter region, thereby transcriptionally activating *BNIP3* expression. Furthermore, we observed a significant increase in the LC3B II/LC3B I ratio, a marker of autophagic activity, and enhanced co-localization of LC3B with TOMM20 (a mitochondrial outer membrane protein) and mitochondrial markers. These findings suggest that gemcitabine treatment may activate mitophagy in NPC cells.

Interestingly, *FOXD1* knockdown not only restricted BNIP3-mediated mitophagy but also increased the sensitivity of NPC cells to gemcitabine. Moreover, inhibiting mitophagy with BNIP3 shRNA further enhanced the inhibitory effects of gemcitabine on cell growth, migration, and invasion in HONE1 cells with high *FOXD1* expression. Glycosylation modification is a prominent feature of malignant tumors and plays a crucial role in regulating protein function. It is involved in various cellular processes, including peptide chain folding, polymerization, transport, and maturation. Glycosylation modifications also contribute significantly to tumor proliferation, invasion, and metastasis 37, 38. *N*-glycosylation modification is a specific type of co-translational or post-translational modification of newly synthesized proteins. This modification involves the attachment of a sugar chain to the free amino group (NH_2_) of a specific asparagine residue (N-X-S/T, where X is any amino acid except proline) within the protein sequence. PNGase F (glycosylpeptidase) is a highly effective glycoside endonuclease that can cleave the glycosidic bond between asparagine residues and *N*-acetylglucosamine. This cleavage removes most *N*-linked sugar chains from glycoproteins, releasing entire oligosaccharide chains 39. Endo H has a narrower substrate specificity, primarily targeting high-mannose and hybrid-type *N*-glycans. This enzyme specifically hydrolyzes the glycosidic bond between *N*-acetylglucosamine residues 39. To investigate the role of glycosylation in FOXD1 function, we treated NPC cells with TM, PNGase F, and Endo H. Western blot analysis confirmed *N*-glycosylation modification of the FOXD1 protein. Subsequently, online prediction tools were utilized to identify three potential *N*-glycosylation sites within the human FOXD1 protein sequence. Through directed mutagenesis, we further confirmed that asparagine residue 176 (Asn-176) serves as the *N*-glycosylation modification site for human FOXD1. A growing body of research highlights the critical role of *N*-glycosylation modifications in the proper folding, functional localization, intracellular transport, and stability of proteins 40.

For our investigation of FOXD1 *N*-glycosylation, we selected two NPC cell lines, HONE1 and C666-1, as primary models. These cell lines were chosen due to their high FOXD1 protein expression and the presence of detectable *N*-glycosylation modifications, as evidenced by the distinct banding patterns observed upon endoglycosidase digestion. These features make them ideal for studying *N*-glycosylation modifications in FOXD1. We treated NPC cells with tunicamycin, PNGase F, and Endo H to investigate the impact of *N*-glycosylation on FOXD1 function. Our findings suggest that *N*-glycosylation modifications can promote the nuclear translocation of FOXD1 and enhance its ability to bind to target DNA sequences, thereby upregulating the transcription of the downstream mitophagy-related gene *BNIP3*. To further explore the *N*-glycosyltransferases involved in FOXD1 glycosylation, we analyzed the expression and prognostic data of 114 glycosyltransferases in NPC tissues. This analysis identified ALG3 as a potential candidate. ALG3 is highly expressed in NPC tissues and exhibits a negative correlation with poor patient prognosis. Functionally, *ALG3* possesses 1,3-glucosyltransferase activity, initiating the biosynthesis of N-linked oligosaccharides within the endoplasmic reticulum, a crucial step in *N*-glycosylation. Mutations in ALG3 can lead to a loss of mannose residues from the oligosaccharide chain 41. Previous work by Sun et al. demonstrated that ALG3 could activate TGFβ signaling by mediating *N*-glycosylation modification of TGFBR2-β, ultimately promoting radioresistance and stem cell characteristics in breast cancer 41. In our study, we observed that overexpression of ALG3 increased the level of *N*-glycosylation modification on FOXD1. This modification, in turn, promoted malignant phenotypes in NPC cells, including enhanced proliferation, migration, and invasion.

This study identified *FOXD1* as a novel factor promoting NPC progression and resistance to gemcitabine therapy. We demonstrate that *FOXD1* was upregulated in NPC tissues and was positively associated with decreased patient survival. Furthermore, our findings elucidated the functional role and underlying mechanism of *FOXD1* in NPC development. We provided hitherto undocumented evidence that gemcitabine-induced mitophagy, a cellular process for eliminating damaged mitochondria, is suppressed by *FOXD1* or *BNIP3* knockdown. FOXD1 could promote cytoprotective mitophagy through transcriptional upregulation of *BNIP3*, thereby facilitating NPC cell migration and invasion, potentially alleviating cellular stress, and ultimately contributing to gemcitabine resistance. Mechanistically, we revealed a novel regulatory pathway where the *N*-glycosyltransferase ALG3 could interact with FOXD1, inducing its *N*-glycosylation and enhancing its nuclear distribution and transcriptional activity. *FOXD1*, along with its upstream regulators and downstream targets, holds promise as both a prognostic marker and a potential therapeutic target for NPC. Our findings suggest that designing inhibitors targeting the ALG3-FOXD1-BNIP3 axis or mitophagy represents a promising therapeutic approach to improve the efficacy of gemcitabine treatment for NPC.

## Conclusions

Collectively, our findings demonstrate that the ALG3-FOXD1 axis enhances mitophagy by transcriptionally activating *BNIP3* expression, thereby promoting NPC progression and modulating the response of NPC cells to gemcitabine. Combinations of therapeutic strategies that target both the ALG3-FOXD1-BNIP3 axis and gemcitabine-induced mitophagy may represent an effective treatment approach for NPC.

## Supplementary Material

Supplementary figures and table.
