# Supplementary material for: Targeting ALG3/FOXD1/BNIP3 Axis Prevents Mitophagy and Gemcitabine Resistance of Nasopharyngeal Carcinoma
Source: Int J Biol Sci. 2025 Feb 10;21(5):1894–913. doi: 10.7150/ijbs.101585 (PMC11900802; doi:10.7150/ijbs.101585)
Supplement: Supplementary file 1 — Supplementary figures and table. [file ijbsv21p1894s1.pdf]

Supplementary Materials

Supplementary Figure S1-13

Supplementary Table S1

Supplementary Figure 1

A

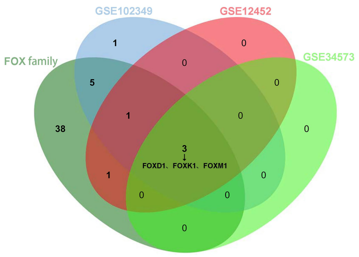

B

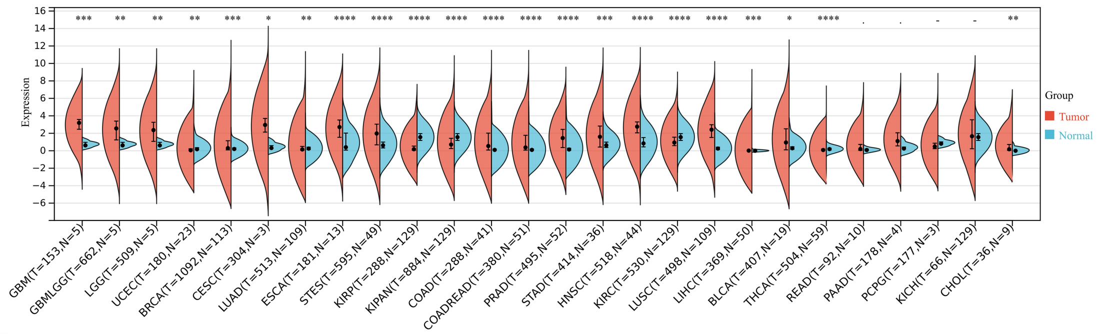

Supplementary Fig. 1. FOX family expression and prognosis in different datasets. A Venn plots show intersection of FOX transcription factors associated with differential expression and survival prognosis in NPC GEO datasets (GSE102349, 113 patients with NPC). **B** Expression of *FOXD1* in different tumors of TCGA.*p < 0.05, **p < 0.01, ***p < 0.001.

Supplementary Figure 2

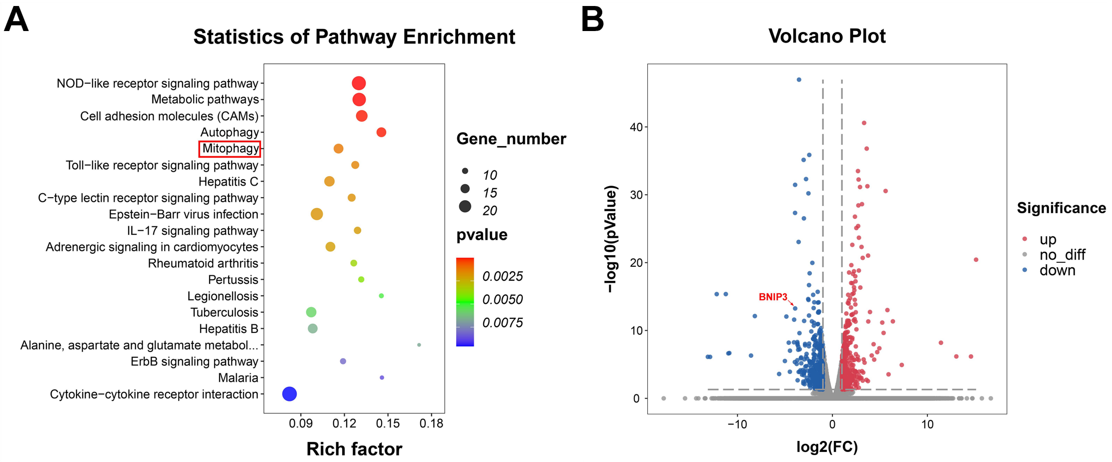

Supplementary Fig. 2. Differentially expressed genes after knockdown of *FOXD1*. A KEGG enrichment analysis of downstream differential genes and pathway after knockdown of *FOXD1*. **B** Volcano plot of differentially expressed genes after knockdown of *FOXD1*.

Supplementary Figure 3

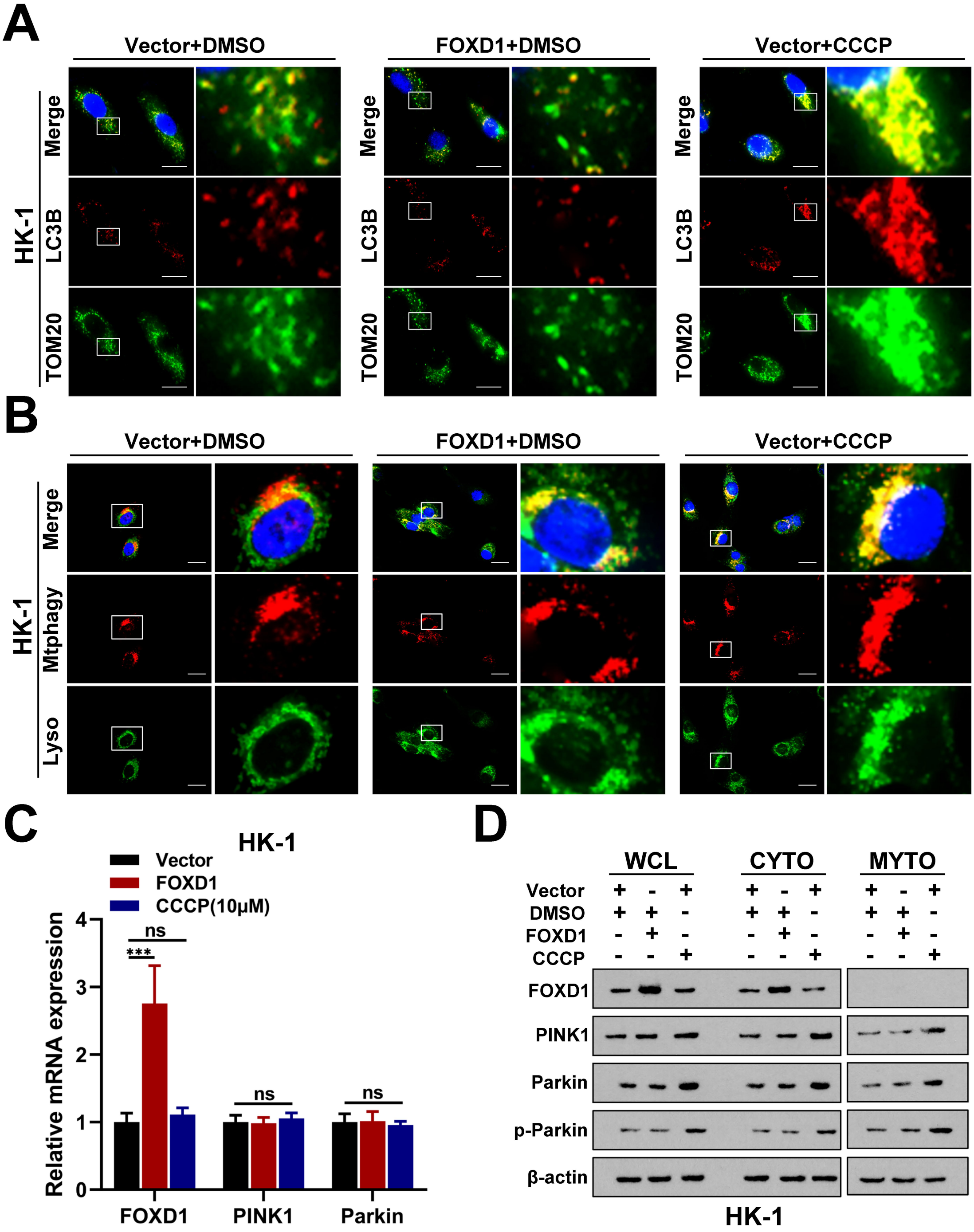

Supplementary Fig. 3. PINK1/Parkin pathway is not the way of FOXD1 inducing mitophagy in NPC. A Fluorescence co-localization experiments showed that the co-localization of TOM20 and LC3B could be increased by overexpression of *FOXD1* or CCCP treatment. **B** Mtphagy Dye and Lyso Dye assay showed that overexpression of FOXD1 or CCCP could promote the fusion of mitochondria and lysosomes in HK-1 cells. **C** qPCR assay showed that overexpression of *FOXD1* could not increase the mRNA expression levels of PINK1 and Parkin compared with control groups. **D** WB assay showed that compared with the control group, the expression levels of PINK1, Parkin and p-Parkin proteins in WCL, CYTO and MYTO in CCCP treated group were

increased to varying degrees. Overexpression of *FOXD1* had no effect on the protein contents of PINK1, Parkin and p-Parkin in each component. ***$p < 0.001$. Scale bars: 10μm.

Supplementary Figure 4

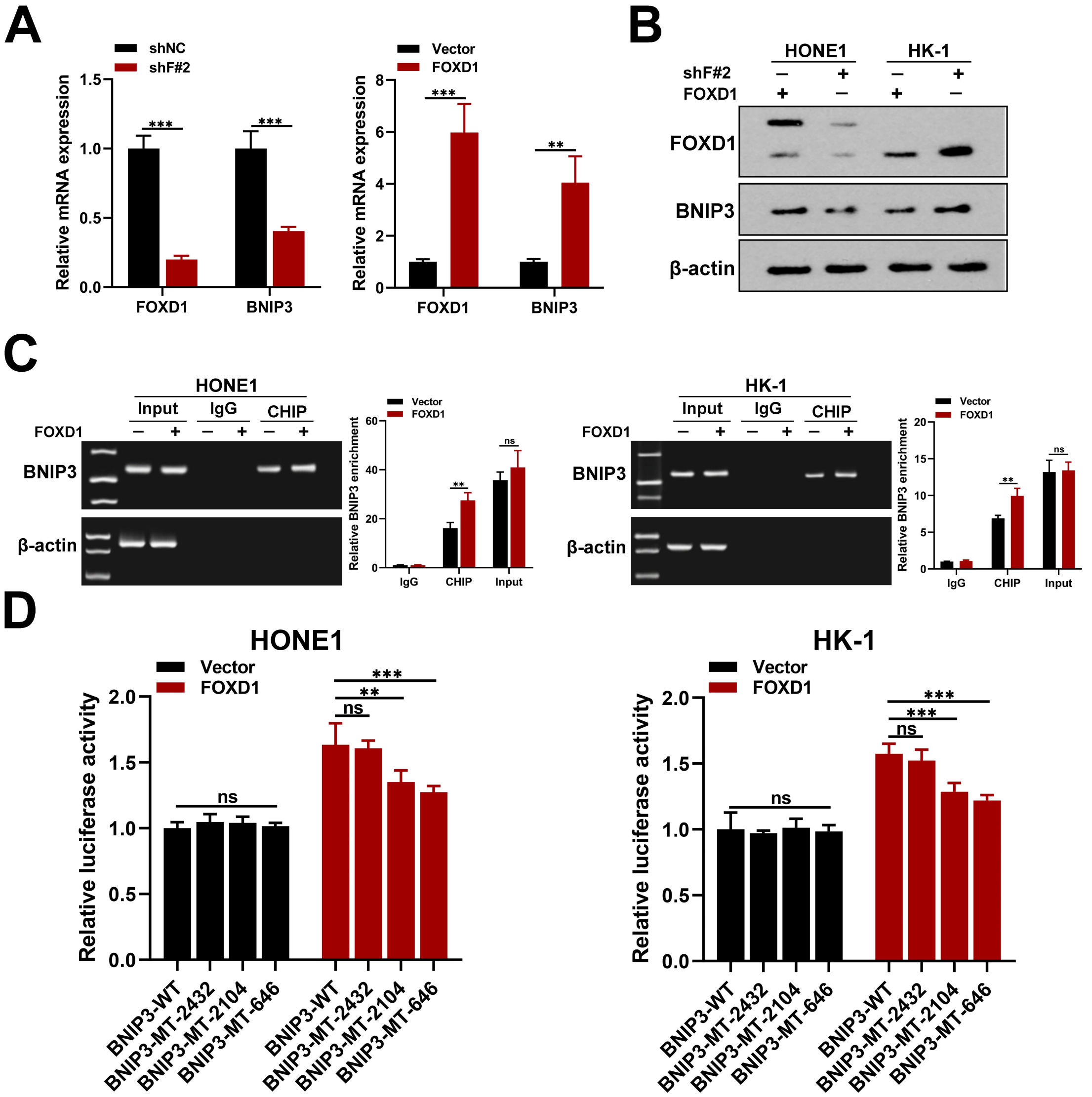

Supplementary Fig. 4. FOXD1 could directly target and transcriptionally up-regulate *BNIP3* expression. Silencing of FOXD1 down-regulated the expression level of *BNIP3* and overexpression of *FOXD1* up-regulated the expression level of BNIP3 by qPCR (**A**) and WB (**B**) assays. **C** CHIP asssay showed that FOXD1 could directly bind to the promoter region of *BNIP3*. **D** The results of dual-luciferase reporter assay showed that FOXD1 could bind to the -2111~-2104bp and -653~-646bp sites in the upstream promoter region of *BNIP3*.

Supplementary Figure 5

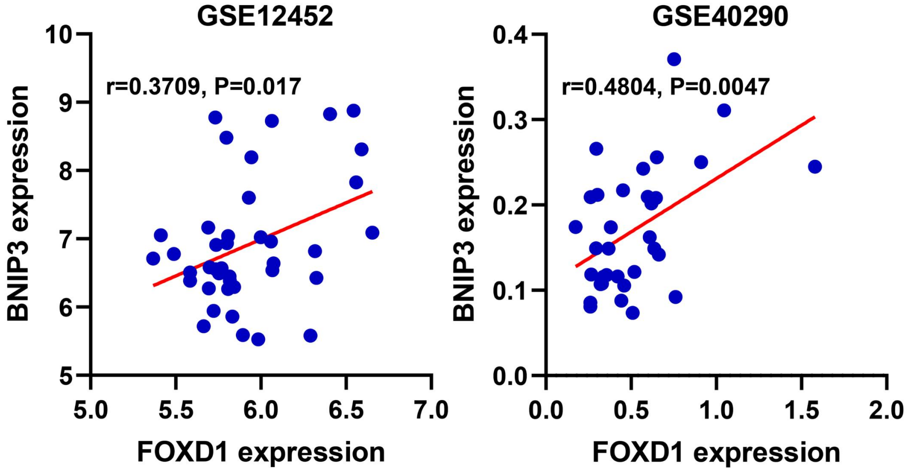

Supplementary Fig. 5. The mRNA expression of *FOXD1* and *BNIP3* was significantly positively correlated in NPC GEO datasets GSE12452 (n=41) and GSE40290 (n=33).

Supplementary Figure 6

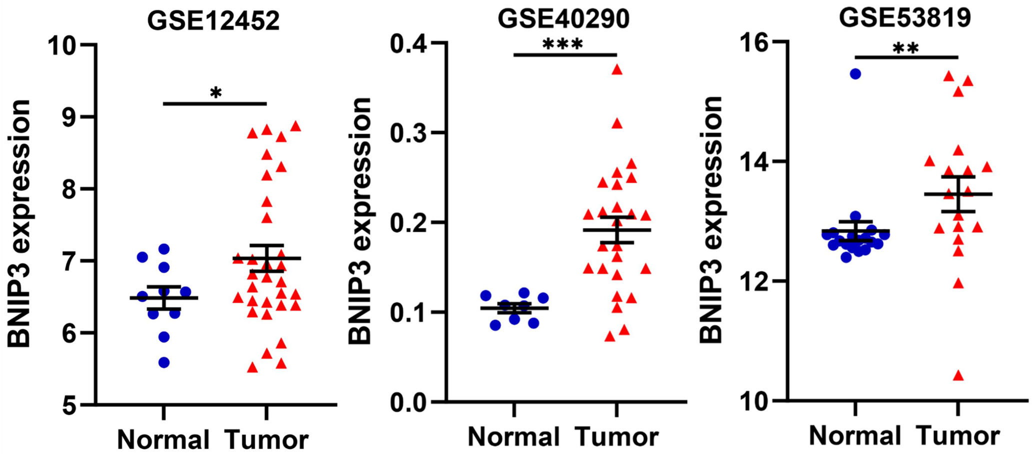

Supplementary Fig. 6. The mRNA expression level of *BNIP3* in NPC GEO datasets (GSE12452, GSE40290 and GSE53819).

Supplementary Figure 7

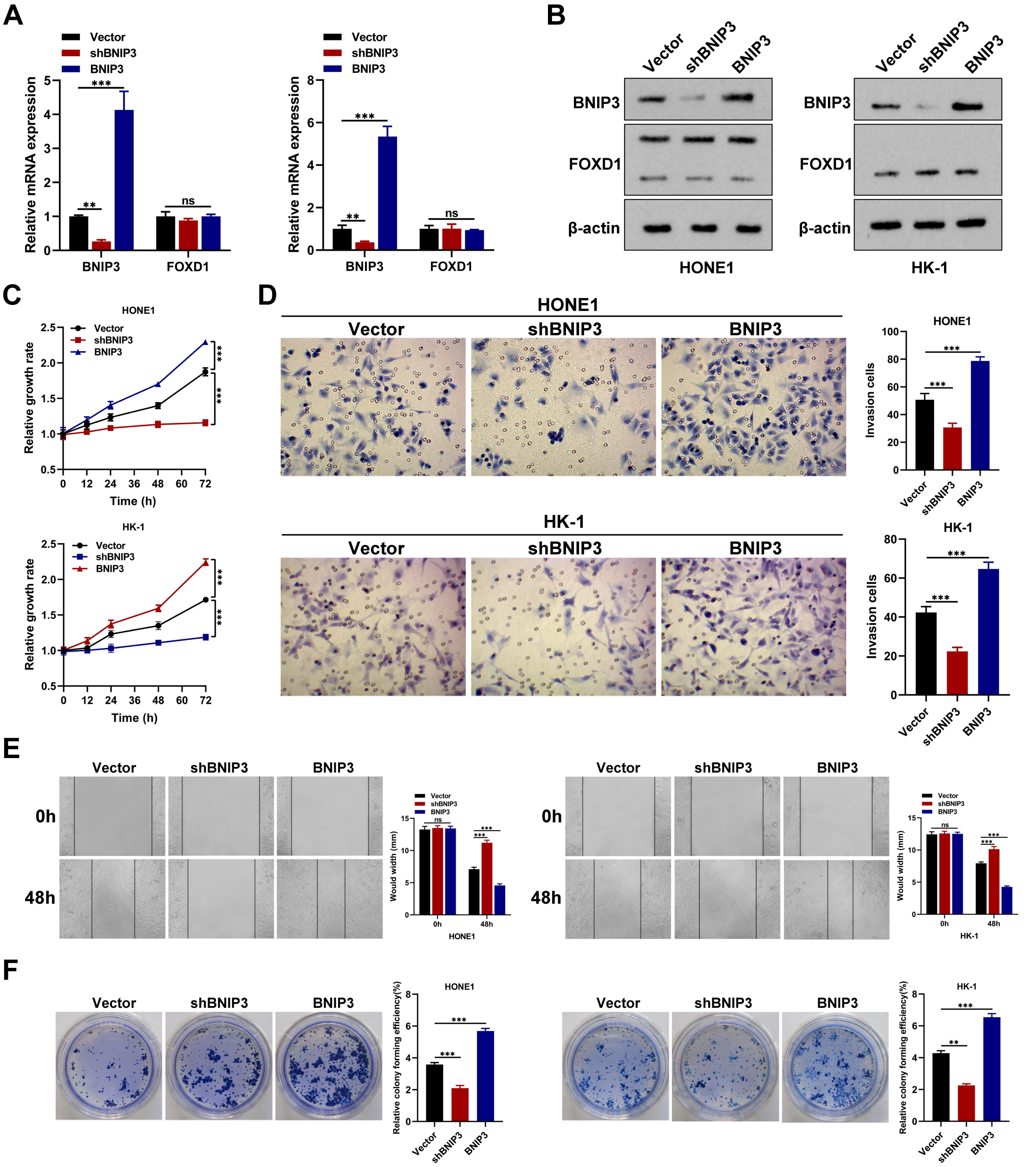

Supplementary Fig. 7. **BNIP3 promoted the biological function of NPC cells in vitro.** The knockout (HONE1 cells) and overexpression (HK-1) efficiency of *BNIP3* was detected by qRT-PCR (**A**) and WB (**B**) assays. CCK8 assay, Transwell assay, wounding healing assay, and colony formation assay were used to measure the cell growth (**C**), invasion (**D**), migration (**E**), and colony forming (**F**) ability after knockdown and overexpression of *BNIP3* in HONE1 and HK-1 cells. **$p < 0.01$, ***$p < 0.001$.

Supplementary Figure 8

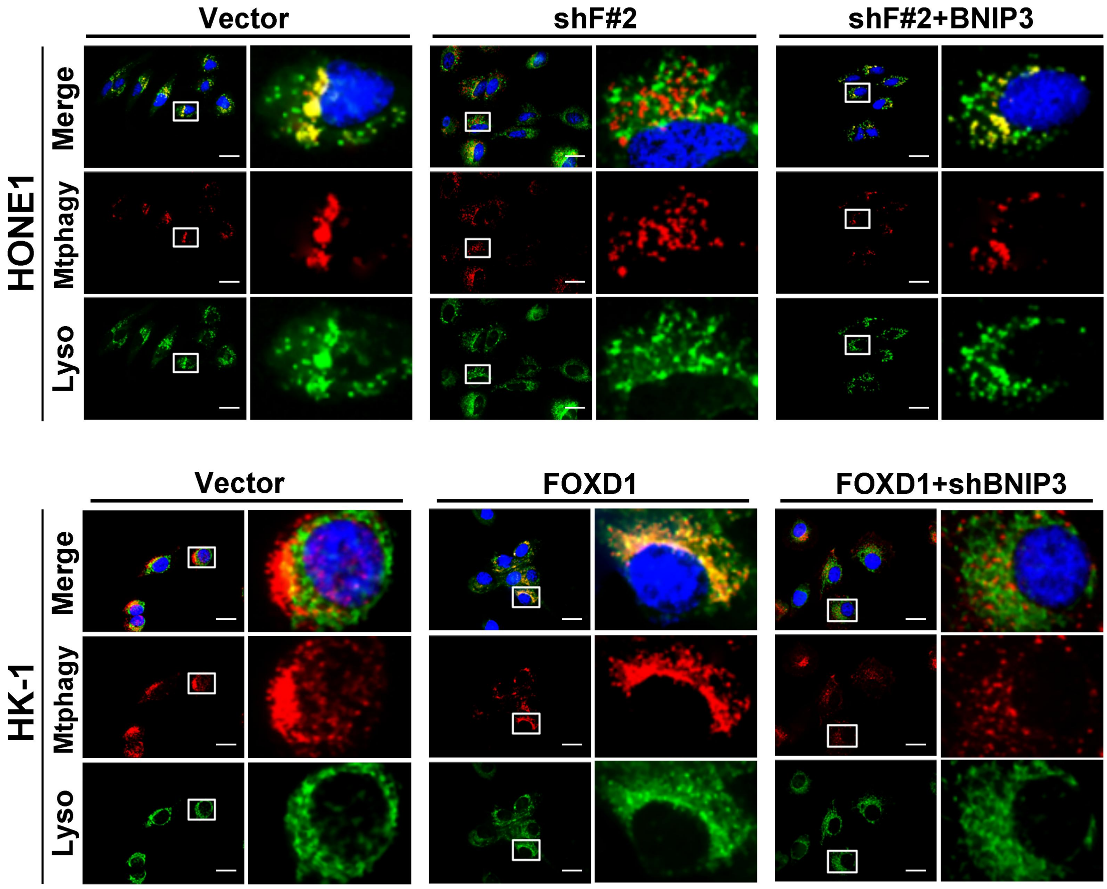

Supplementary Fig. 8. Mtphagy Dye and Lyso Dye assay demonstrated that FOXD1 enhance mitophagy level of NPC cells through BNIP3. Scale bars: 10μm.

Supplementary Figure 9

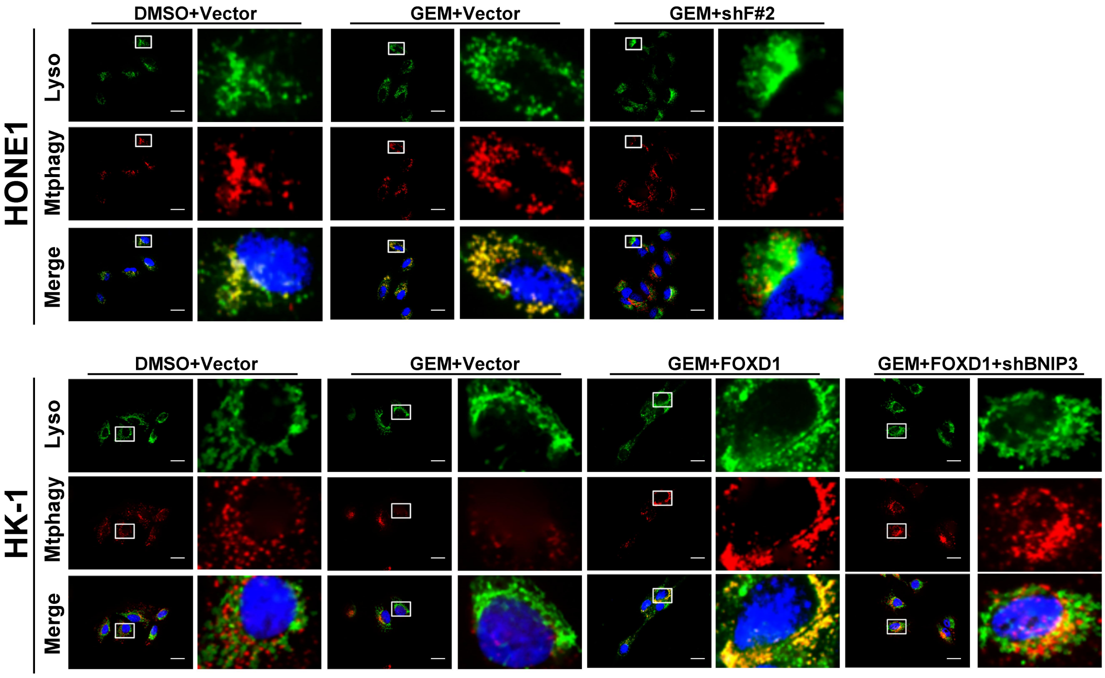

Supplementary Fig. 9. Mtphagy Dye and Lyso Dye staining assay demonstrated that *FOXD1* could enhance the effect of GEM on mitophagy level of HONE1 and HK-1 cells. Scale bars: 10μm.

Supplementary Figure 10

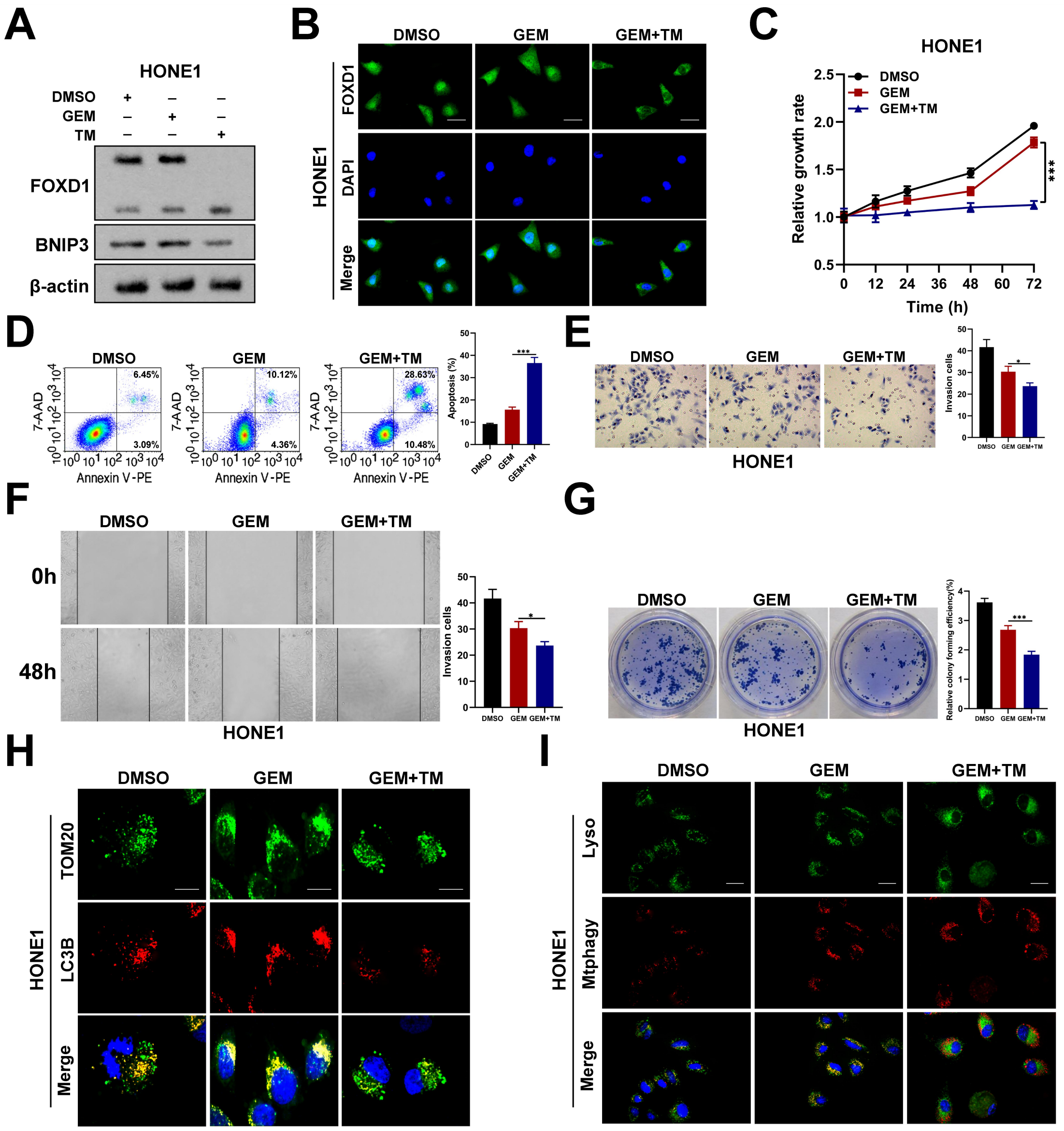

Supplementary Fig. 10. **N-glycosylation modification of FOXD1 impair the drug sensitivity of NPC cells to GEM. A** TM treatment could lead protein bands of FOXD1 migrate downward. **B** IF assay confirmed that TM treatment could significantly reduced the nuclear localization of FOXD1. CCK8 assay, flow cytometry, Transwell assay, wounding healing assay, and colony formation assay were used to measure the cell growth (**C**), apoptosis (**D**), invasion (**E**), migration (**F**) and colony forming (**G**) ability with or without TM treatment in HONE1 cells. TOM20 and LC3B fluorescence colocalization assay (**H**), as well as Mtphagy Dye and Lyso Dye assay showed that GEM could improve the mitophagy level, while the addition of TM could reduce the mitophagy level of NPC cells. Scale bars: 10μm.

Supplementary Figure 11

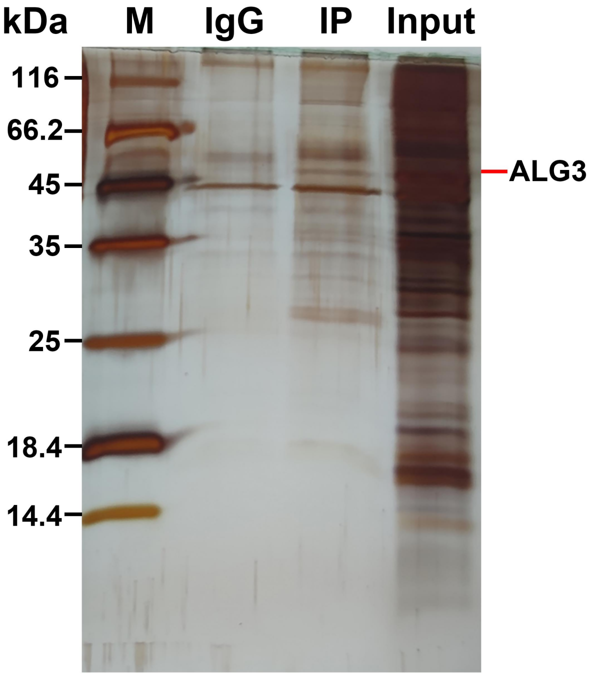

Supplementary Fig. 11. co-IP and silver staining assay showed that FOXD1 protein could directly bind with ALG3 protein.

Supplementary Figure 12

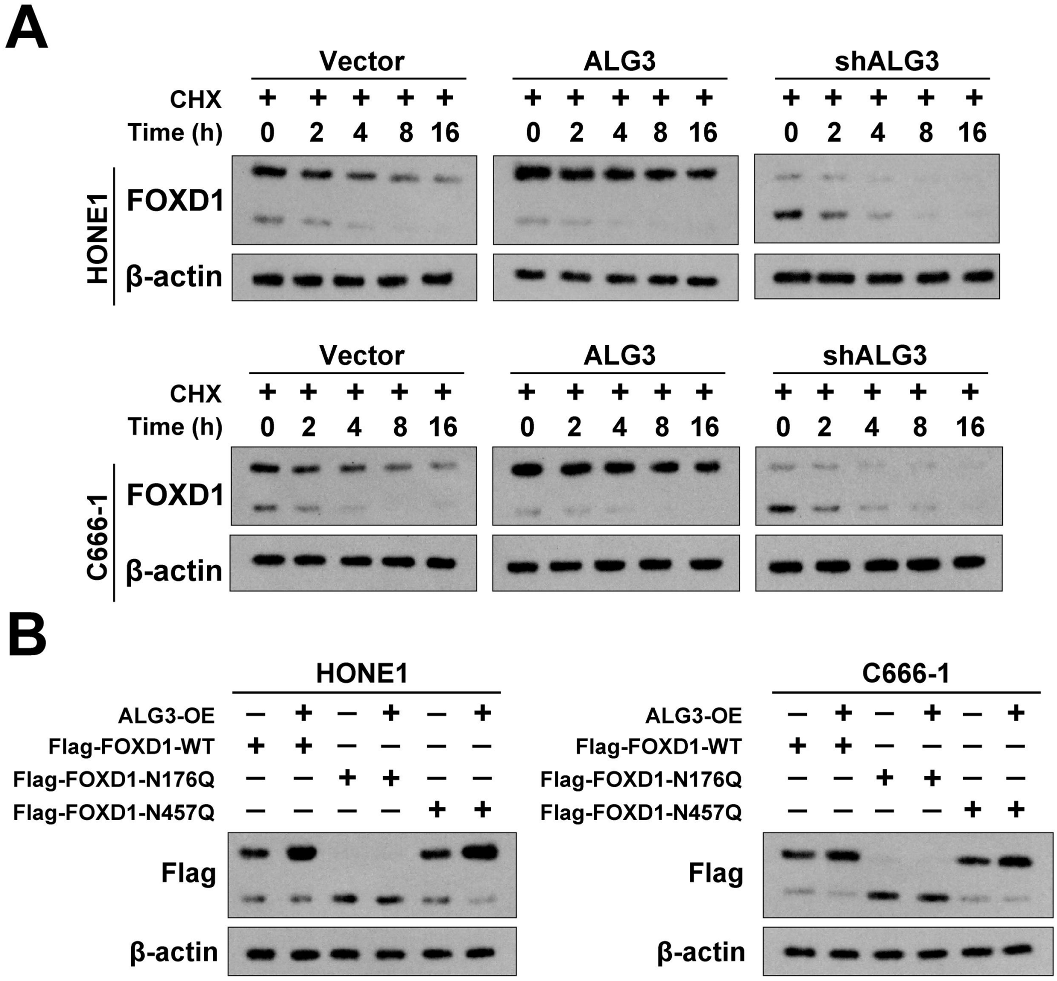

Supplementary Fig. 12. ALG3 enhanced the protein stability of FOXD1 through mediating the *N*-glycosylation of FOXD. **A** CHX ans WB assays in HONE1 and C666-1 cells revealing that ALG3 inhibited the degradation of FOXD1 protein. **B** WB assay was performed to detect the expression of Flag protein in HONE1 and C666-1 cells transfected with FLAG-FOXD1-WT, FLAG-FOXD1-N176Q and FLAG-FOXD1-N457Q plasmid combined with or without ALG overexpression plasmid.

Supplementary Figure 13

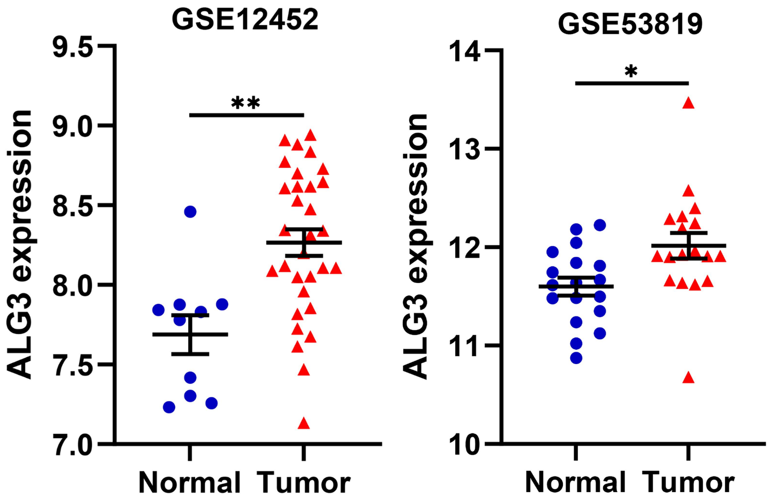

Supplementary Fig. 13. *ALG3* is highly expressed in NPC tissues (GSE12453 and GSE53819).

Supplementary Table S1

The sequences of primers and shRNA used in this study

**The sequences of primers for PCR (5′→3′)**	
FOXD1	Forward: TGAGCACTGAGATGTCCGATG
	Reverse: CACCACGTCGATGTCTGTTTC
BNIP3	Forward: TGAGTCTGGACGGAGTAGCTC
	Reverse: CCCTGTTGGTATCTTGTGGTGT
ALG3	Forward: CCGAGGTAGAAGGCGTCATC
	Reverse: GGTACACAAGTGGTCCGGT
PINK1	Forward: GCCTCATCGAGGAAAAACAGG
	Reverse: GTCTCGTGTCCAACGGGTC
parkin	Forward: CCCACCTCTGACAAGGAAACA
	Reverse: TCGTGAACAAACTGCCGATCA
β-actin	Forward: ACCCTGAAGTACCCCATCGAG
	Reverse: AGCACAGCCTGGATAGCAAC
**The targeted sequences of shRNAs (5′→3′)**	
shFOXD1#1	TACTGCTAGGATTTCCAATTGTTAA
shFOXD1#2	GCCGAGGAAACAGACATCGACGTGG
shFOXD1#3	GAGCACTGAGATGTCCGAT
shBNIP3	CGTTCCAGCCTCGGTTTCTATTTAT
shALG3	GGTTTCGTGTACATCTTTATG
